# Supplementary material for: Extravascular gelation shrinkage-derived internal stress enables tumor starvation therapy with suppressed metastasis and recurrence
Source: Nat Commun. 2019 Nov 26;10:5380. doi: 10.1038/s41467-019-13115-3 (PMC6879564; doi:10.1038/s41467-019-13115-3)
Supplement: Supplementary file 1 — Supplementary Information [file 41467_2019_13115_MOESM1_ESM.pdf]

Supporting information

Extravascular gelation shrinkage-derived internal stress enables tumor starvation therapy with suppressed metastasis and recurrence

Kun Zhang et al

Parts of experimental section

Materials

All starting materials were analytical grade from commercial sources and used without further purification. Cetyltrimethylammonium bromide (CTAB), sodium borohydride ($NaBH_4$), potassium carbonate (K_2CO_3), ascorbic acid, tetrachloroauric acid ($HAuCl_4 \cdot 3H_2O$), silver nitrate ($AgNO_3$), and hydrochloric acid (HCl, 37 wt % in water) were purchased from Sinopharm Chemical Reagent Co., Ltd. HS-PEG-SH (MW=20 kDa) were purchased from Seebio Biotech (Shanghai) Co., Ltd. (Shanghai, P. R. China). Soluble carboxylated chitosan, mPEG-Mal (MW=20 KDa) and acrylic acid (AAc, GC, ＞99.7%) were purchased from Aladdin Biotech. CO., LTD (China, Shanghai). N-isopropylacrylamide (NIPAAm) and a,a‘-Azobisisobutyronitrile (AIBN) were purchased from Sigma-Aldrich, Co, Ltd. NIPAAm was further purified with benzene/n-hexane by re-crystallization prior to use. Deionized (DI) water (Millipore Milli-Q grade) with a resistivity of 18.2 MΩ was used in all experiments. Diethyl ether, methanol, acetone and acetic acid were obtained from Sinopharm Chemical Reagent Co.,Ltd. Anhydrous ethanol, methanol, iron(III) chloride hexahydrate ($FeCl_3 \cdot 6H_2O$), ammonium acetate (NH_4Ac) and ethylene glycol (EG) were purchased from Sinopharm Chemical Reagent, Shanghai, China. Antibodies against cell surface markers for the flow cytometry assay were purchased from eBioscience. Enzyme-linked immunosorbent assay (ELISA) kit was purchased from R&D Systems, USA. FITC-labeled Dextrans with varied sizes (*i.e.*, 70K, 20K and 4K) and triphenyltetrazolium chloride (TTC) were purchased from Sigma-Aldrich, and Cy3-labeled Dextrans was purchased from Nanocs Co. LTD..

Synthesis of Gold Nanorods

Gold nanorods (GNRs) with an aspect ratio of ~4 were obtained using a well-established seedless

growth method. In detail, 10 mmol CTAB was dissolved in 10 mL of $HAuCl_4$ solution (5 mM), and subsequently the solution was diluted into 100 mL with DI water and vigorously stirred. Afterwards, 120 μL of $AgNO_3$ solution (0.1 M) was dropwise added and the reaction proceeded for 5 min. Immediately afterwards, 600 μL of ascorbic acid (AA) solution (0.1 M) was added above solution to reduce Au (III) to Au (I), followed by injecting 40 μL of $NaBH_4$ solution (0.1 M) to promote nucleation and growth, and the growth process lasted for another 4 h. The CTAB-capped GNRs were collected *via* high-speed centrifugation at 12000 rpm for 15 min. Furthermore, the obtained GNRs were repeatedly washed *via* dispersion in DI water under ultrasonication and centrifugation at 12000 rpm for 15 min.

Synthesis of PEG-coated GNRs (GNR-PEG-SH)

The surfaces of the as-prepared CTAB-capped GNRs were modified with thiol-modified PEG (HS-PEG-SH) *via* a classic ligand-substitution method. Typically, 2 mM K_2CO_3 and 1 mM HS-PEG-SH aqueous solutions were added to the aforementioned CTAB-capped GNRs solution at a volume ratio of 1:0.2:10, respectively. After 24 h incubation at room temperature, GNR-PEG-SH was harvested and repeatedly washed *via* dispersion in DI water and high-speed centrifugation under at 12,000 rpm for 15 min. Ultimately, the collected GNR-PEG-SH was dispersed into DI water and kept at 4 °C for use. The absorbance of the GNR solution was measured using a Shimadzu 3101PC UV/Vis spectrophotometer. The atomic concentration of GNRs was quantified by an inductively coupled plasma-atomic emission spectrometer (ICP-AES, Agilent). FETEM (field emission transmission electron microscopy) analysis was conducted with a JEM 2100 F electron microscope operated at 200 kV to characterize the PEG-coated GNRs. Scanning electron microscopy (SEM) images/scanning transimission electron microscopy (STEM) images and corresponding element

mapping were obtained on a field emission Maggellan 400 microscope (FEI Company).

Synthesis of linear pNIPAAm-*co*-AAc coploymers

Linear pNIPAAm-*co*-AAc copolymers were yielded *via* a well-established *in-situ* free radical copolymerization method using NIPAAm and AAc as the raw materials. Typically, in a three-necked, round-bottomed flask, 44.25 mmol of NIPAAm was dissolved in 90 mL of methanol along with a certain amount of AAc in sequence, followed by with ultrasonic agitation for 5 min. The polymerization that was accelerated by AIBN initiator was carried out under N_2 atmosphere at 60 °C and lasted for 24 h, during which continuous stirring was always accompanied. Afterwards, the resultant copolymers were harvested *via* the solvent extraction-mediated precipitation method after adding excessive diethyl ether, and subsequently they were further rinsed by dissolution in acetone and precipitation in excessive diethyl ether for three cycles. Ultimately, the obtained pNIPAAm-*co*-AAc copolymers were dried in vacuum at room temperature for 24 h prior to further use.

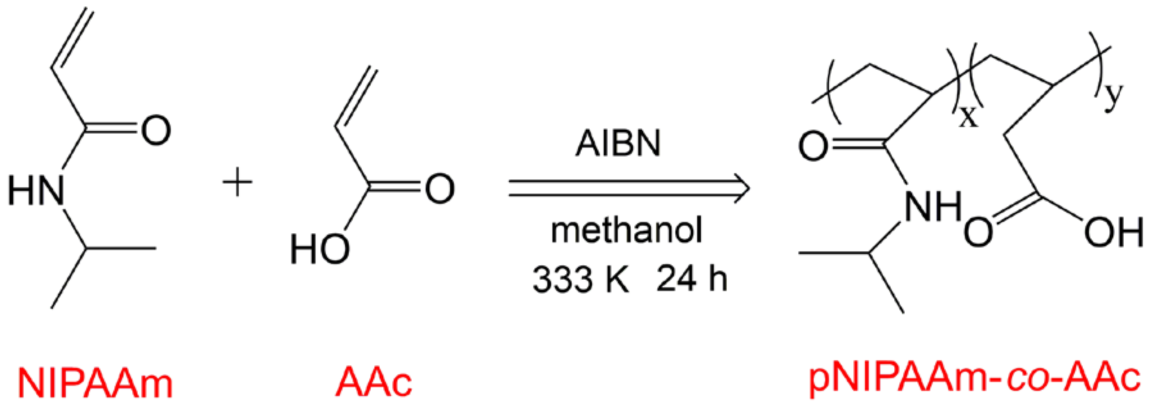

Synthesis of chitosan/mPEG-mal/pNIPAAm-*co*-AAc hydrogel and its derivative (hydrogel-GNR)

Briefly, chitosan/mPEG-Mal/pNIPAAm-*co*-AAc composite hydrogel (0.9 g) at the feed ratio of

20/10/70 was dissolved in 70 mL of acetic acid solution (2% v/v). The solution was shaken for 2 days and was then filtered using a glass funnel to remove undissolved materials, and the ultimate solution was stored at 4 °C for use. As for hydrogel-GNR, GNRs were added in above initial mixture and stirred for 8 h so as to guarantee the chelation of GNRs *via* the thiol–maleimide click reaction between GNR-EPH-SH and mPEG-Mal. Ultimately, the obtained composite solution was stored at 4 °C for use. The pores of as-prepared gel were characterized by scanning electron microscope (SEM) on a field-emission JEOL JSM-6700F microscope. The absorbance of the hydrogel-GNR sol was measured using a Shimadzu 3101PC UV/Vis spectrophotometer.

Synthesis of hollow porous Fe_3O_4 nanoparticles

The magnetic porous hollow Fe_3O_4 nanoparticles were obtained according to a simple solvothermal reaction. In brief, $FeCl_3 \cdot 6H_2O$ (1.350 g) was completely dissolved in 70 mL of EG by magnetic stirring and ultrasonic irradiation, and then NH_4Ac (3.854 g) was added into above mixture for further dissolution. The above mixture was dissolved completely under vigorous magnetic stirring at room temperature for 30 min, and then was transferred into a teflon lining of hydrothermal synthesis reactor (100 mL capacity) and sealed by high pressure-tolerant outer lining made of stainless steel. Afterwards, the high pressure reactor was incubated in a preheated oven at 200 °C for 16.5 h. After cooling the reactor to room temperature, the black HIONs were collected and washed with deionized water, respectively. Size and structure were analyzed *via* Scanning electron microscopy (SEM) on a field emission Maggellan 400 microscope (FEI Company) and transmission electron microscopy (TEM) on a JEM-2100F electron microscope operated at 200 kV were N_2 desorption and adsorption isotherms, Zeta potential, XRD patterns.

Supplementary Figures

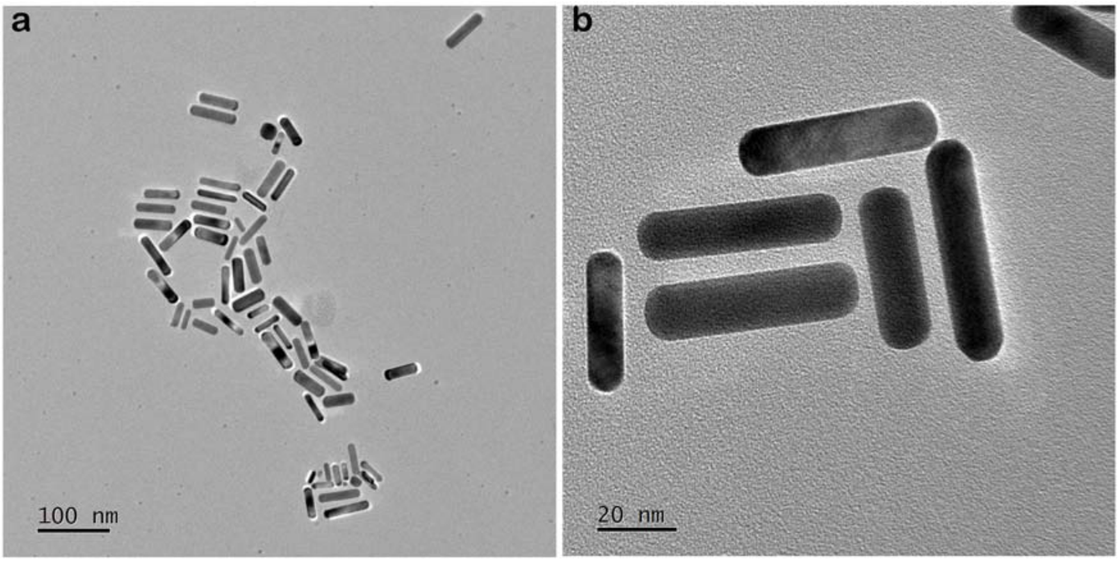

Supplementary Figure 1. Low-fold (a) and high-fold (b) magnification TEM images of GNRs.

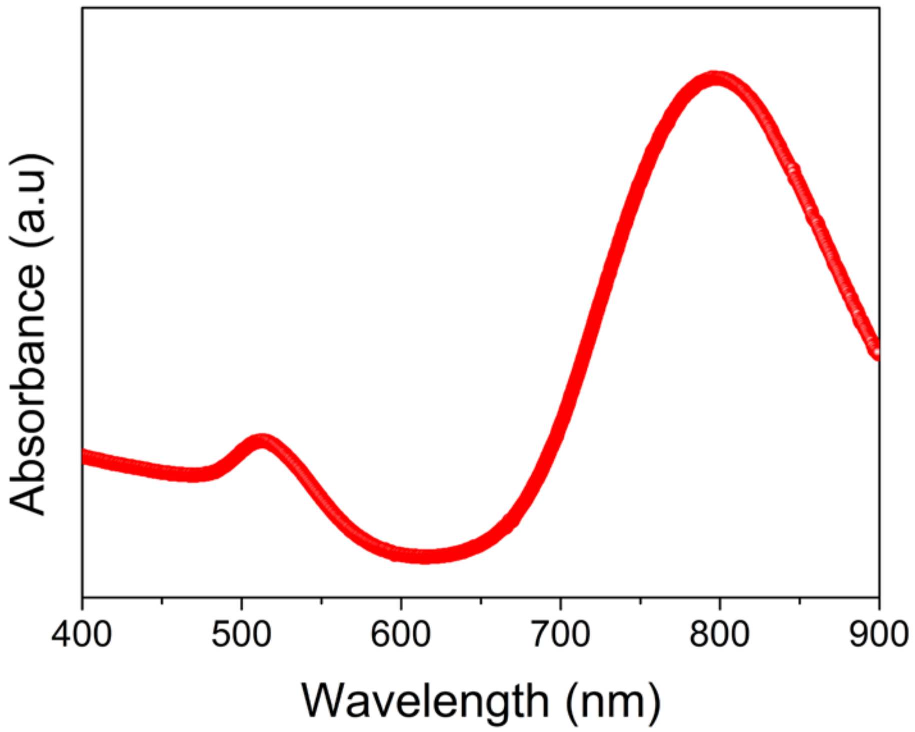

Supplementary Figure 2. UV-vis spectrum of thiol-terminated PEG-modified GNRs (GNR-PEG-SH).

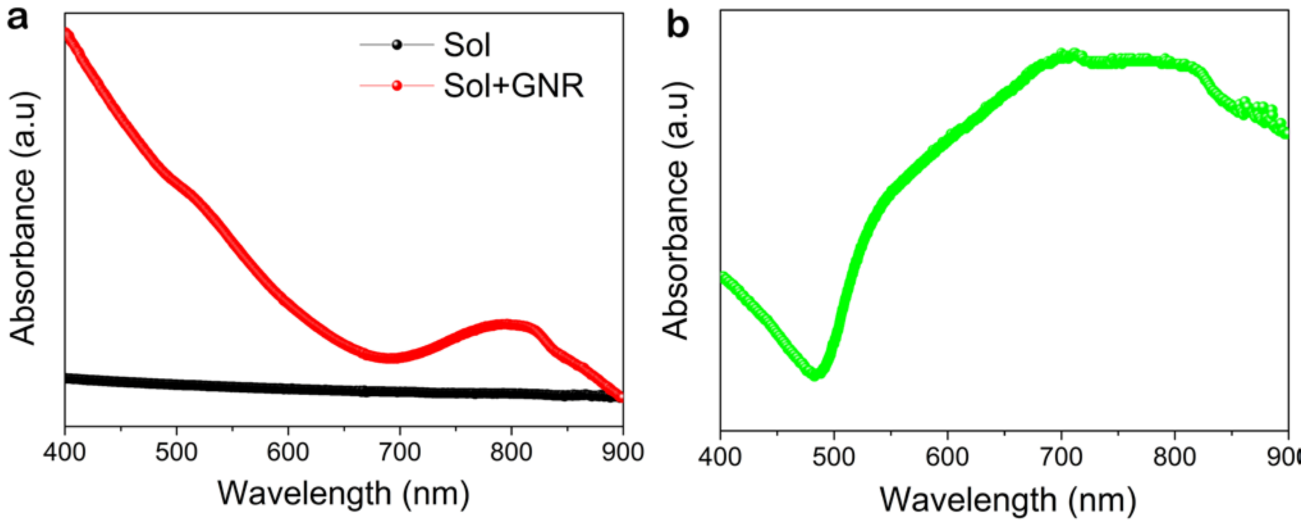

Supplementary Figure 3. UV-Vis spectra of hydrogel and hydrogel-GNR before (a) and after (b) gelation.

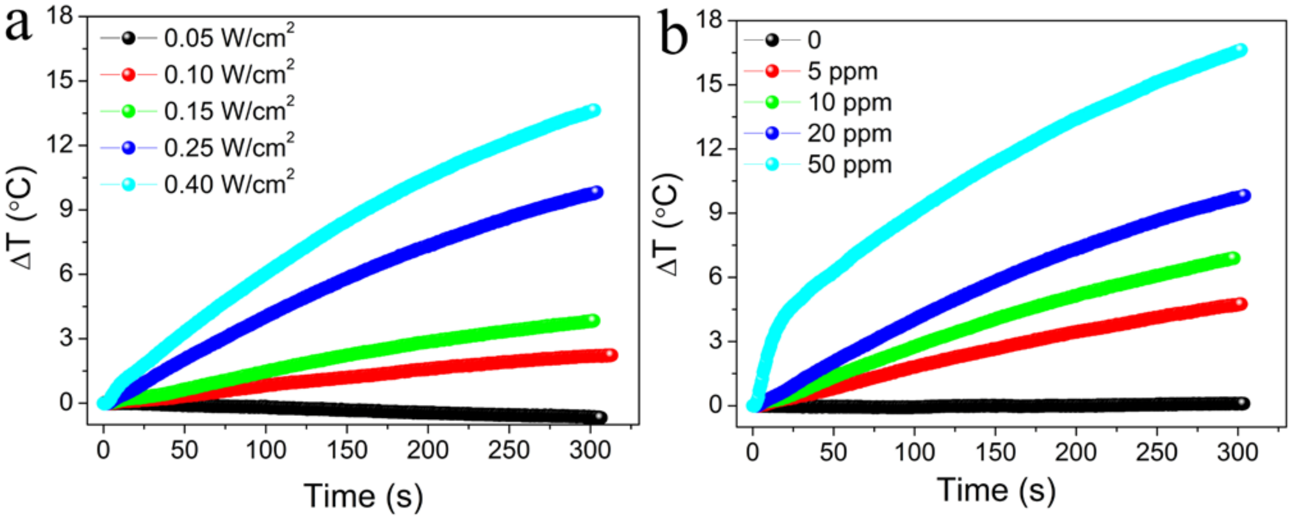

Supplementary Figure 4. *In vitro* time-dependent temperature variation profiles of hydrogel-GNR exposure to different power densities of 808 nm laser (GNRs: 20 ppm) (a) or different concentrations (b) of GNRs (power density: 0.25 W·cm^{-2}).

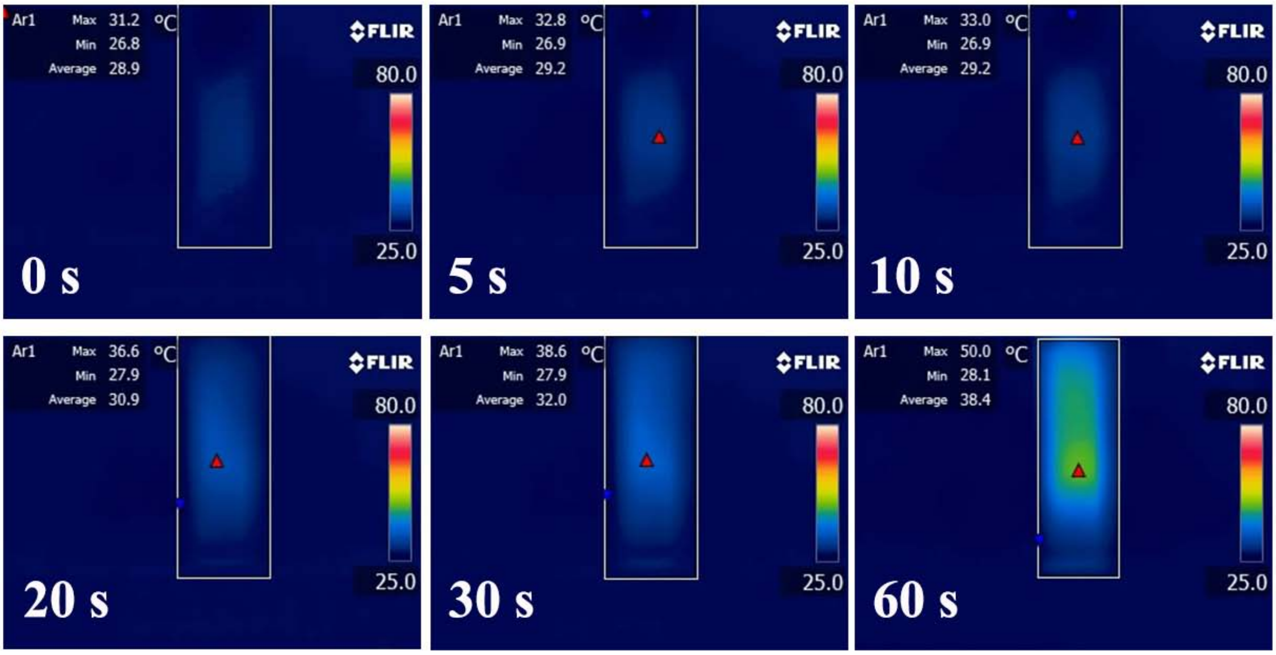

Supplementary Figure 5. *In vitro* thermal infrared images of hydrogel-GNR (20 ppm) at a given power density of 0.15 W·cm^{-2}.

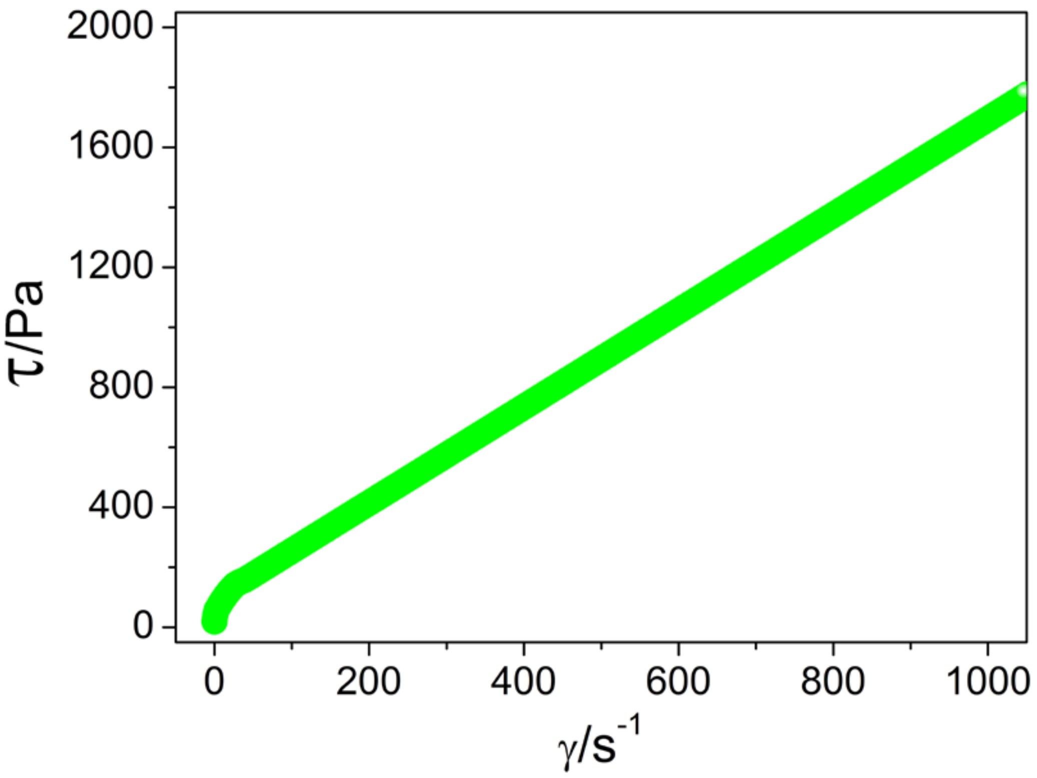

Supplementary Figure 6. Fluid test of hydrogel-GNR that is represented by the relationship between shear stress and shear rate.

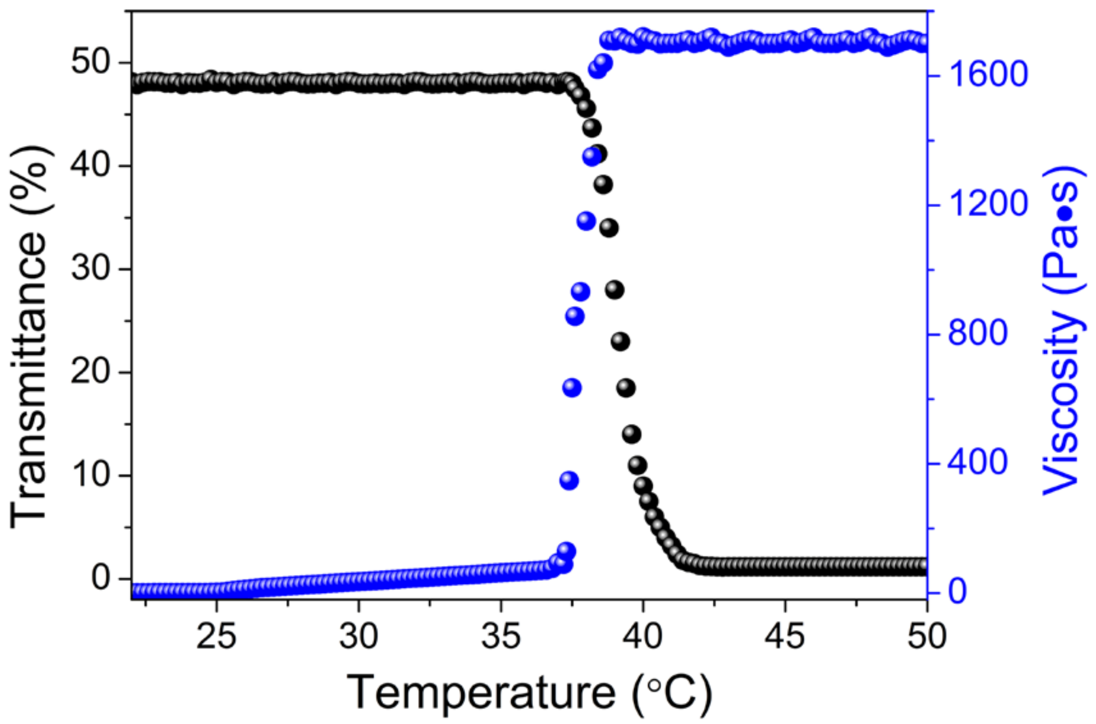

Supplementary Figure 7. Temperature-dependent transmittance and viscosity of hydrogel-GNR.

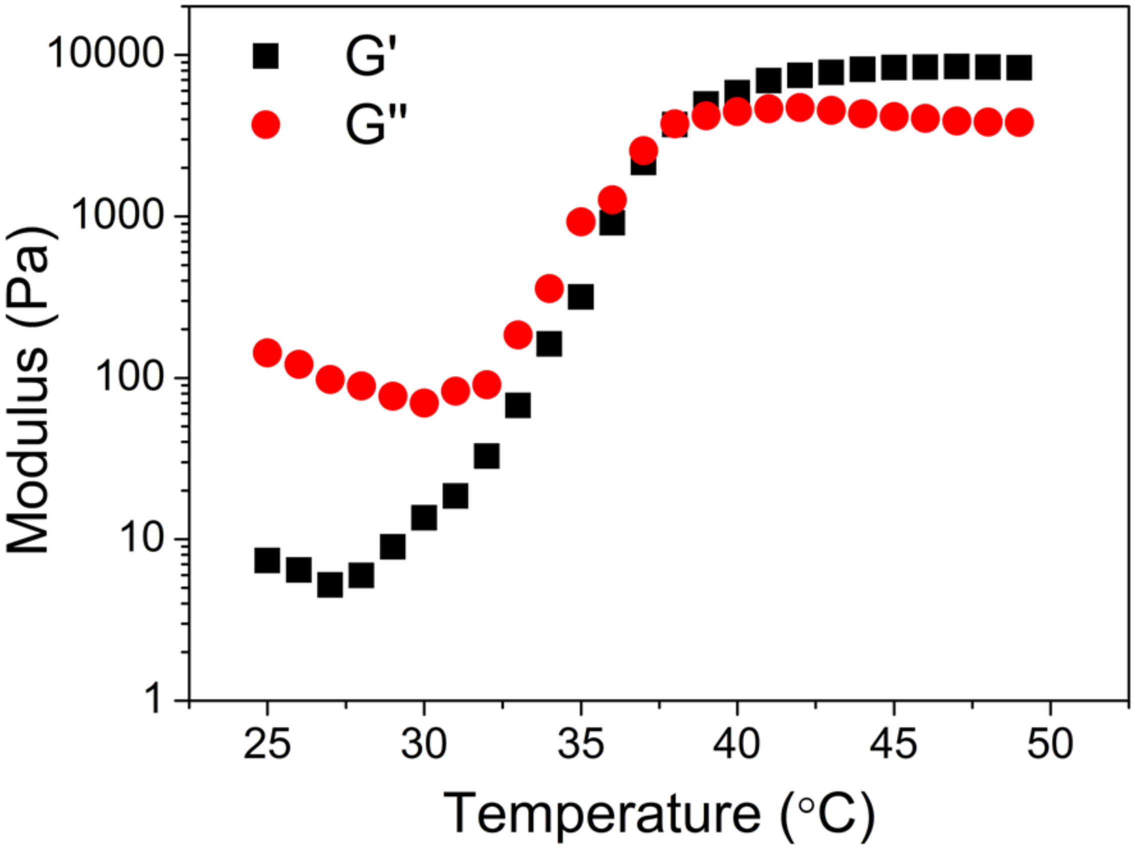

Supplementary Figure 8. Moduli G′ and G″ as a function of temperature for hydrogel-GNR.

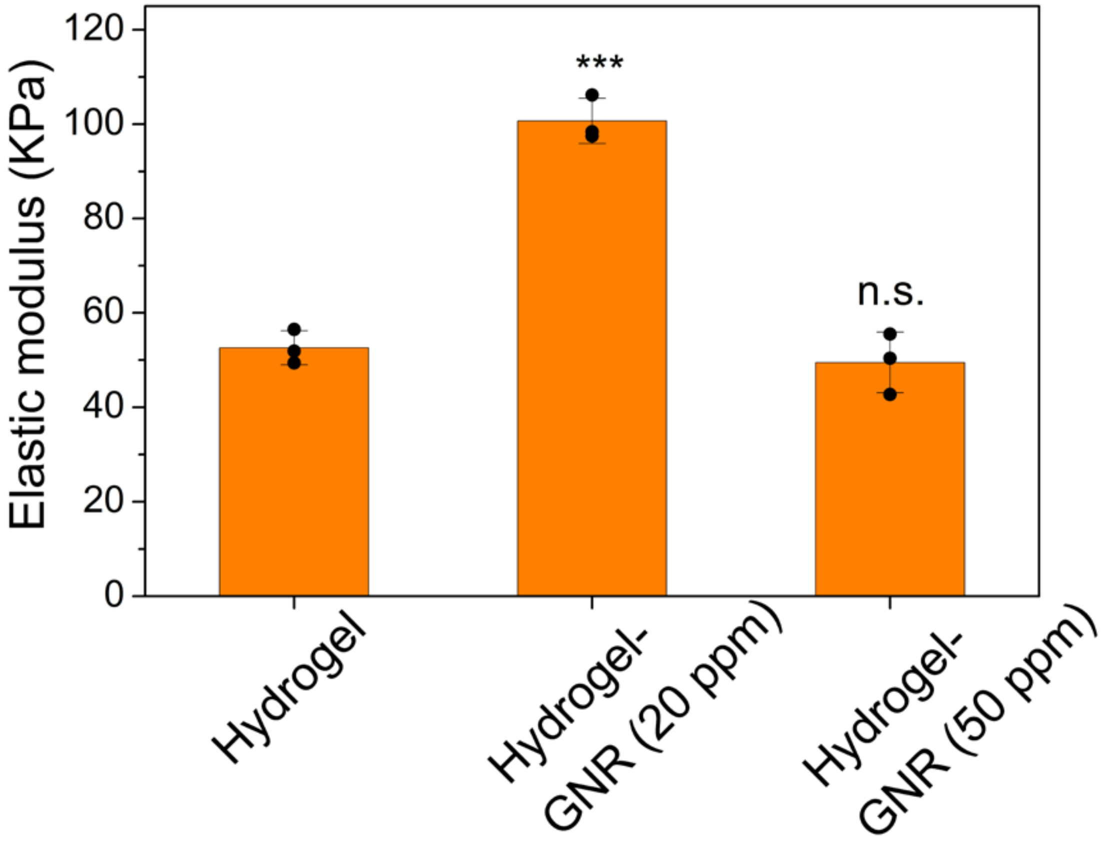

Supplementary Figure 9. Elastic modulus of hydrogel, hydrogel-GNR (20 ppm) and hydrogel-GNR (50 ppm) determined as strain=10%. Values are expressed as mean ± standard deviation (SD) (n = 3). Statistical significance were calculated *via* unpaired Student's t-test in comparison to Hydrogel; 'n.s.', not significant, and ***P<0.001.

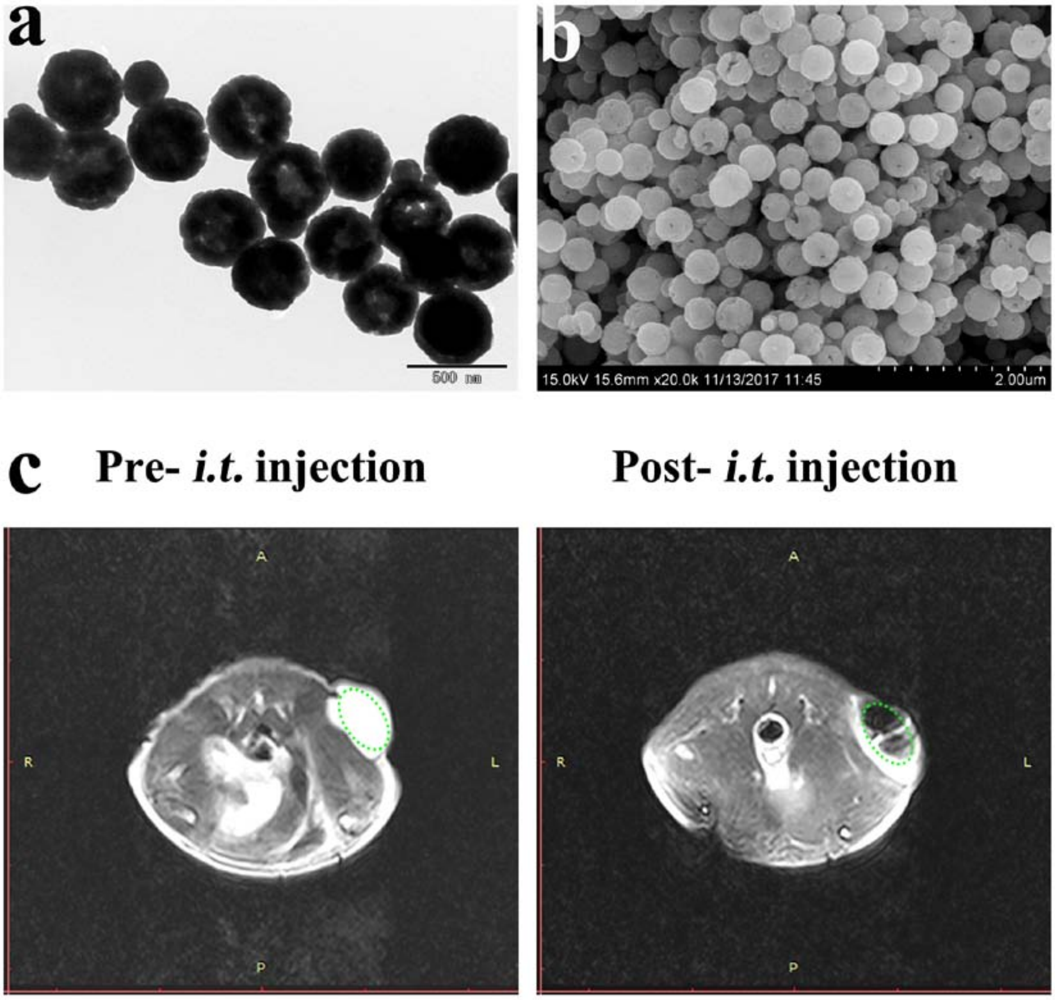

Supplementary Figure 10. (a,b) TEM and SEM images of hollow porous Fe_3O_4 nanoparticles with a diameter of 400 nm; (c) Transversal T2-weighted MRI images of PANC-1 xenografted tumor subcutaneously implanted in nude mice before (left) and after (right) intratumorally injecting hollow porous Fe_3O_4 nanoparticles (2 $mg{\cdot}mL^{-1}$). The instant decrease of MRI signal (that is, positively enhancement) suggest the diffusion of Fe_3O_4 nanoparticles pervaded all around the tumor.

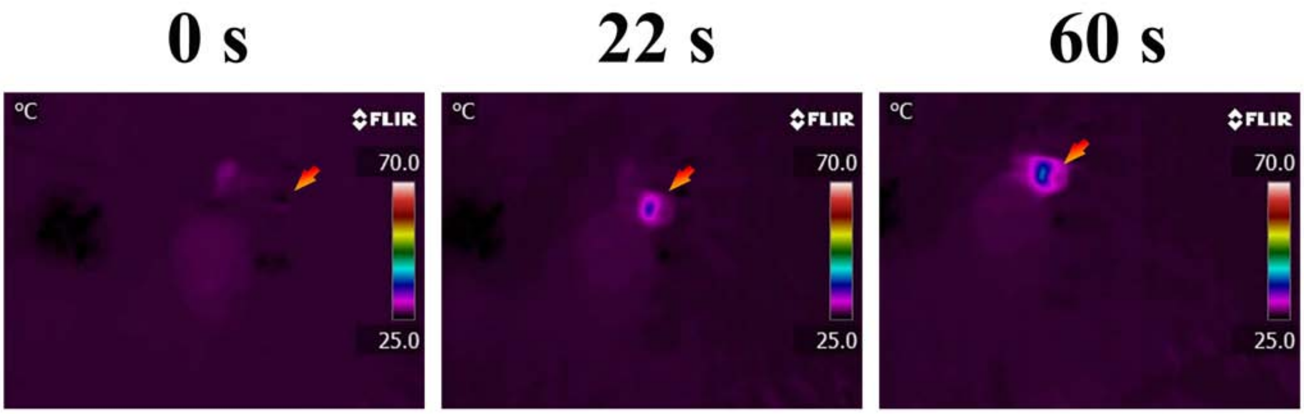

Supplementary Figure 11. Time-dependent thermal infrared images of PANC-1 tumor after injecting hydrogel-GNR and 808 nm laser irradiation with a power density of 0.15 W·cm^{-2}.

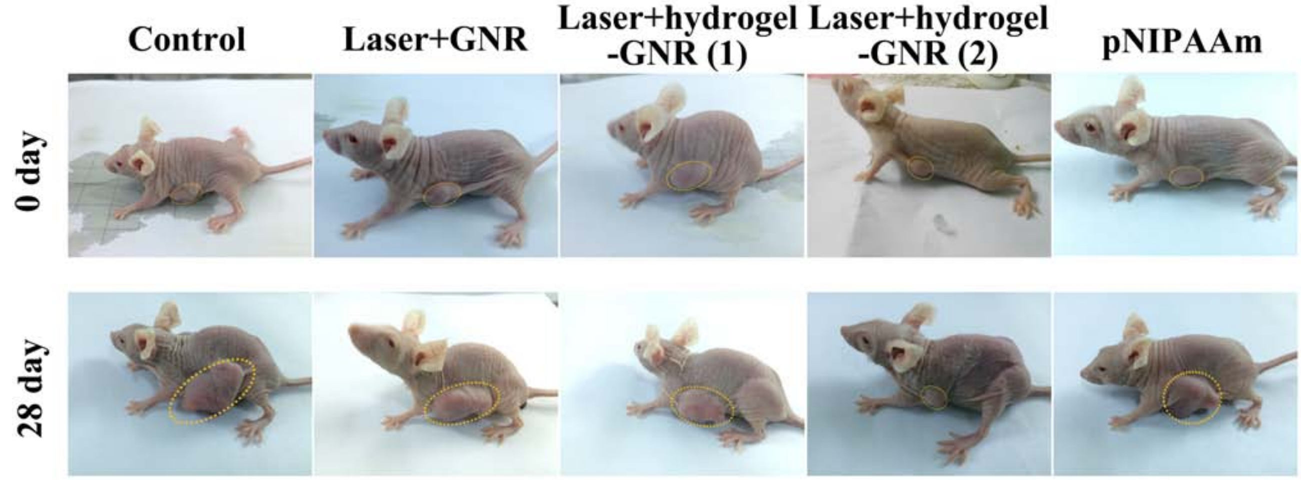

Supplementary Figure 12. Digital photos of nude mice bearing PANC-1 tumor undergoing different treatments at 0 day and 28 day, respectively.

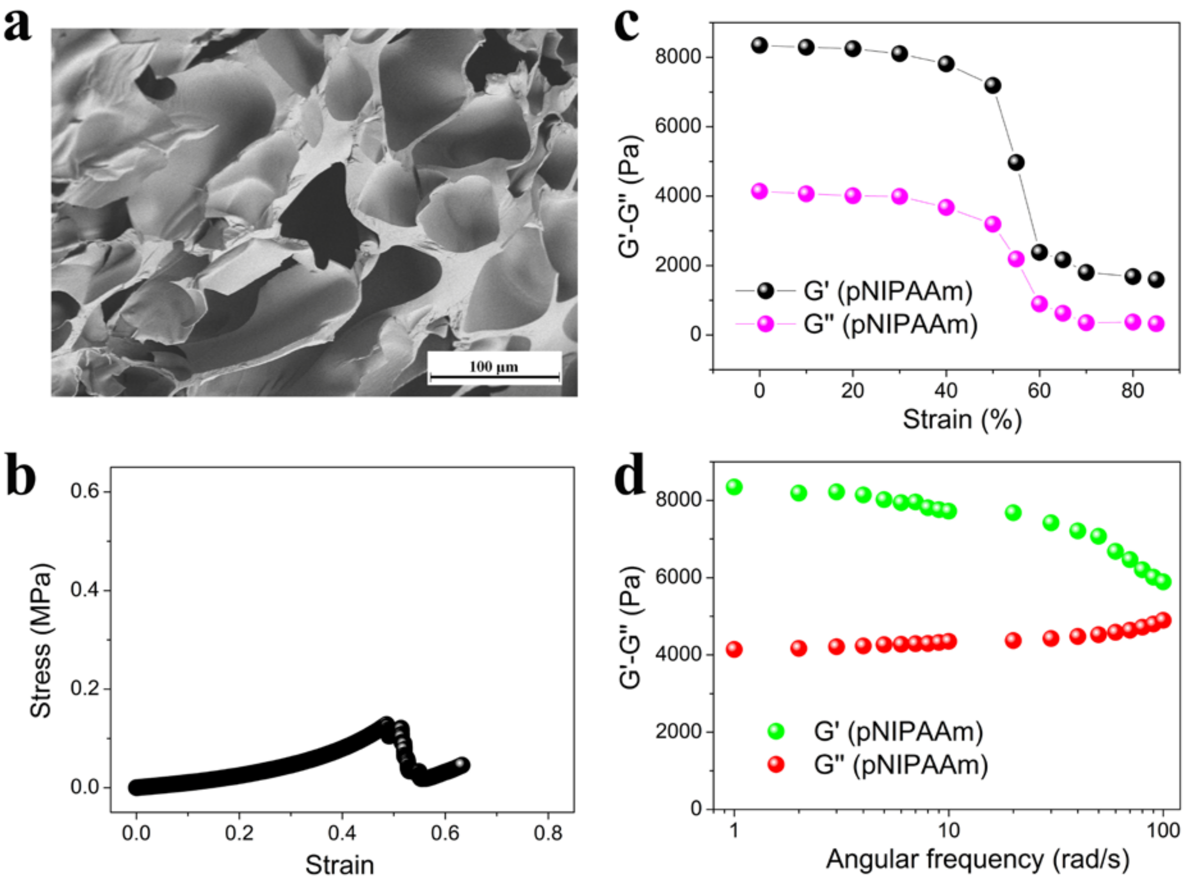

Supplementary Figure 13. SEM image (a), compressive stress-strain curve (b), strain amplitude sweep at 39 °C at a constant frequency of 1 rad·s^{-1} (c) and dynamic oscillatory frequency sweep at a fixed strain of 1% (d) of pNIPAAm.

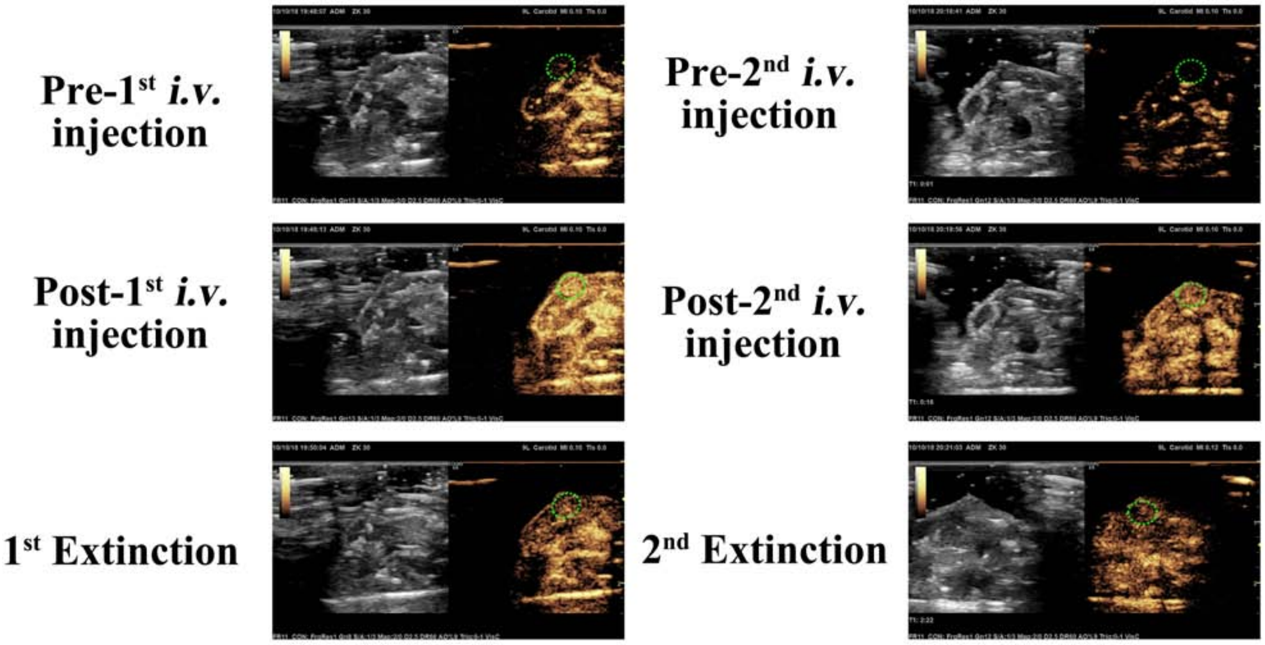

Supplementary Figure 14. Time-dependent ultrasound imaging images of PANC-1 xenografted tumor before and after corresponding treatments in pNIPAAm group, wherein the 1st *i.v.* injection of Sonovue™ microbubbles were carried out, and after 30-90 min, the corresponding treatment (*i.e.*, *i.t.* injecting pNIPAAm) were implemented, followed by the 2nd *i.v.* injection of Sonovue™ microbubbles; Notes: ultrasound images were captured at three time points, *i.e.*, pre-, post- and 40-120 s post- each *i.v.* injection, respectively; and green dotted ellipse indicates the tumor.

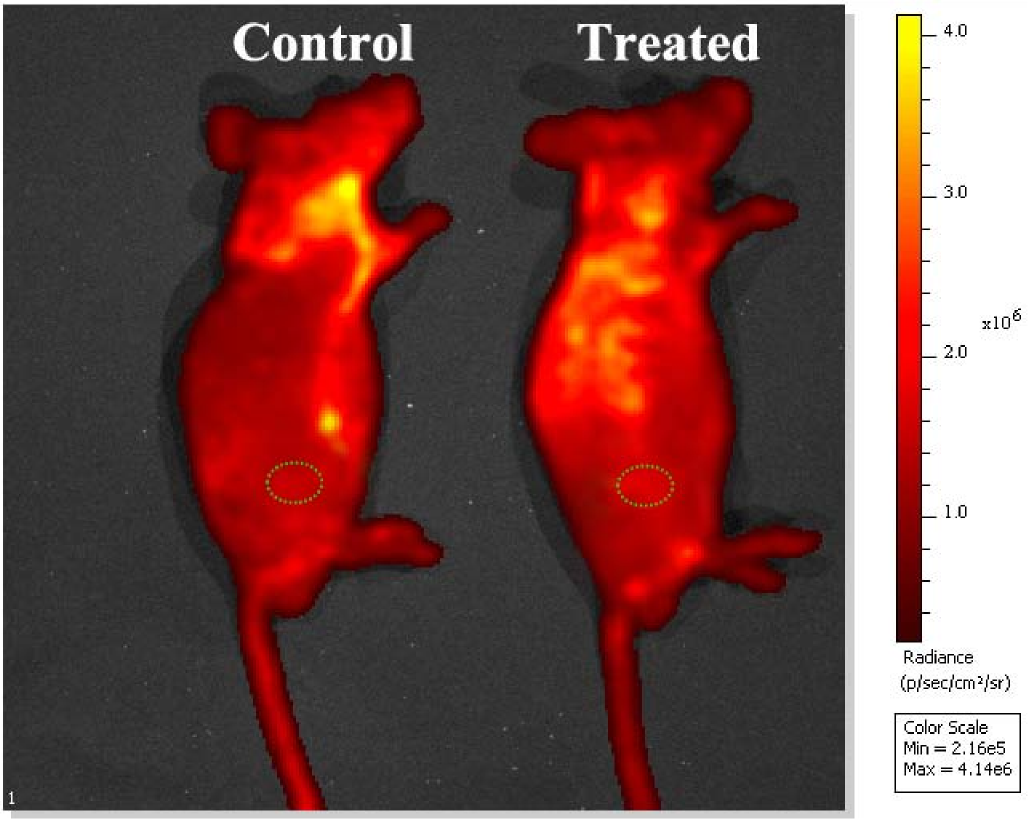

Supplementary Figure 15. *In vivo* animal fluorescence imaging of nude mice bearing PANC-1 xenografted tumor after corresponding treatments in Control and Treated (*i.e.*, pNIPAAm) groups and subsequent injection of different FITC-labeled dextrans with varied sizes (*i.e.*, 70K), wherein green dotted ellipses indicate the tumor

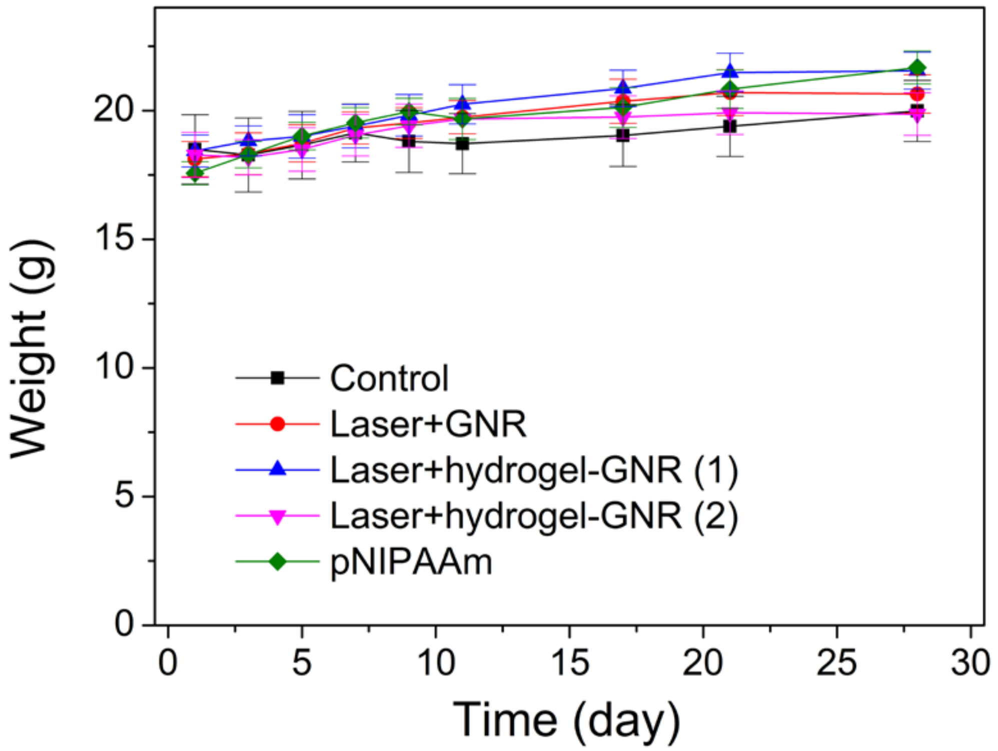

Supplementary Figure 16. Weight fluctuations of nude mice bearing PANC-1 tumor undergoing different treatments from 0 day to 28 day. Data are expressed as mean ± SD (n = 6).

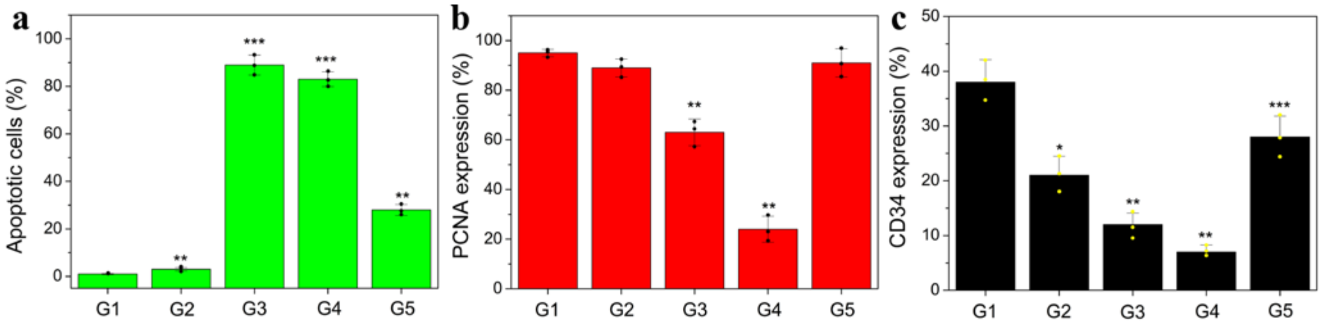

Supplementary Figure 17. Quantitative data of H&E (a), PCNA (b) and CD34 (c) immunochemistry staining against PANC-1 tumor slices after different treatments. *P<0.05, **P<0.01 and ***P<0.001, and the statistical significance were obtained using t-student test in comparison to G1. Data are expressed as mean ± SD (n = 3). G1-G5 represent Control, Laser+GNR, Laser+hydrogel-GNR (1), Laser+hydrogel-GNR (2) and pNIPAAm, respectively.

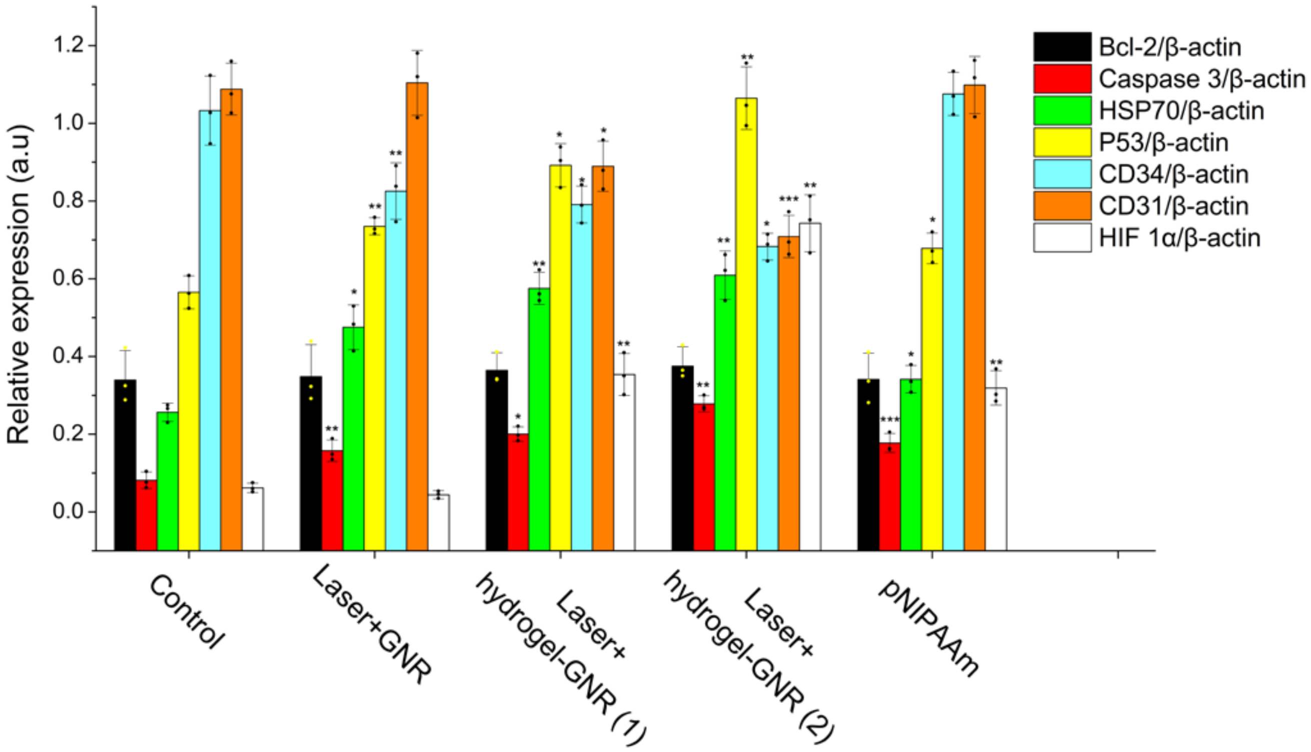

Supplementary Figure 18. Relatively quantitative western blot data of different proteins in tumors treated with different groups. Data are expressed as mean ± SD (n = 3). *P<0.05, **P<0.01 and ***P<0.001, and statistical significance were obtained using t-student test in comparison to Control.

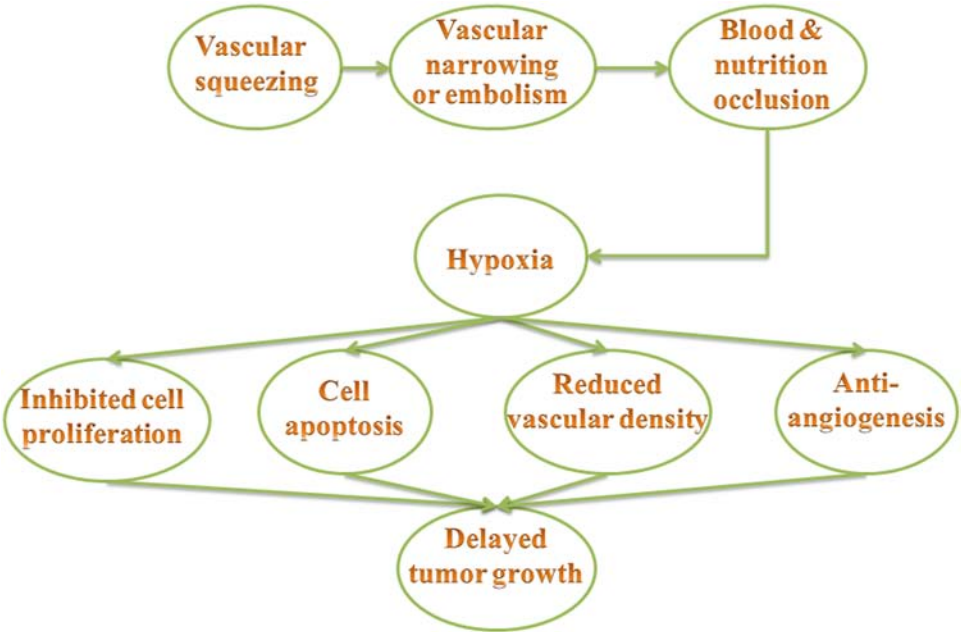

Supplementary Figure 19. The underlying principles of this extravascular gelation shrinkage-derived internal stress for tumor starvation therapy.

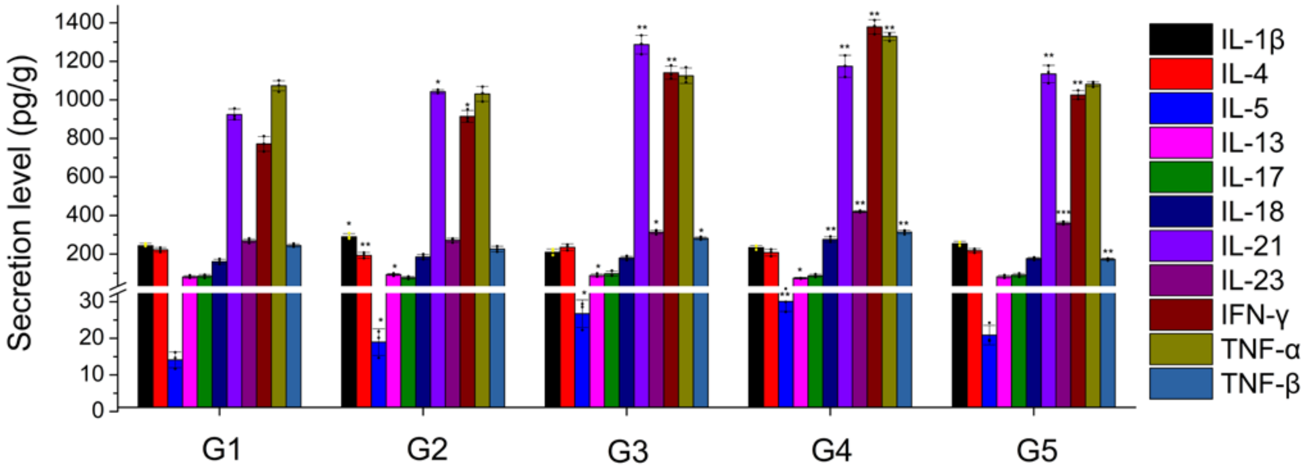

Supplementary Figure 20. Quantitative secretion levels of inflammation factors in tumor after different treatments, *i.e.*, G1-G5 that represent Control, Laser+GNR, Laser+hydrogel-GNR (1), Laser+hydrogel-GNR (2) and pNIPAAm, respectively. Data are expressed as mean ± SD (n = 3). *P<0.05, **P < 0.01 and ***P < 0.001, and statistical significances were obtained using t-student test in comparison to G1.

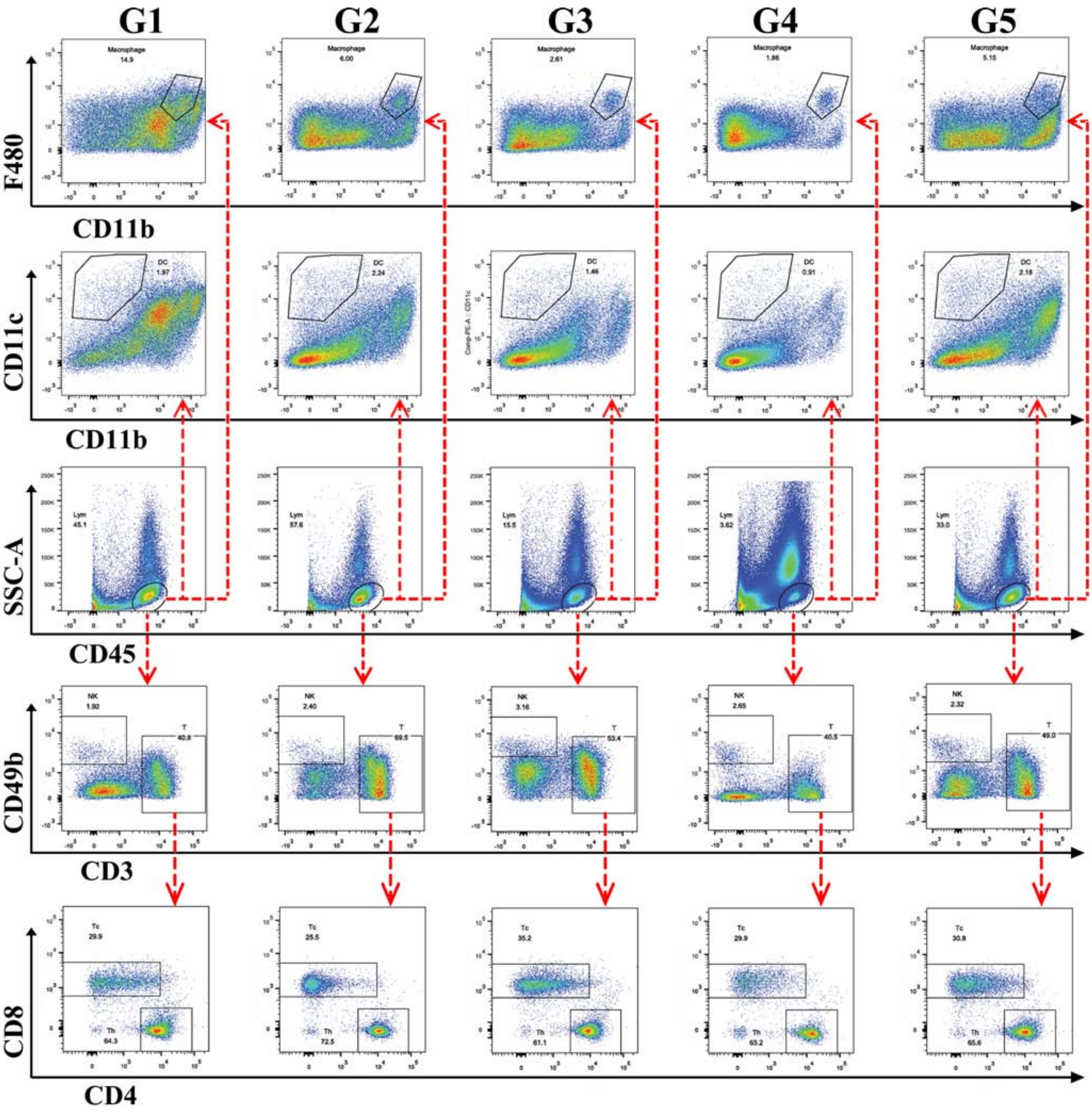

Supplementary Figure 21. Gating strategies used for cell sorting in 4T1 tumors after aforementioned different treatments (G1-G5) at day 10 obtained from the representative flow cytometry plots, in detail, Gating strategy to sort macrophages (CD11b+F480+), dendritic cells (Cd11b-CD11c+), NK cells (Cd3-CD49b+) and T lymphocytes (CD3+CD49b+) from tumor infiltrating leukocytes cells (CD45+) for immune microenvironment analysis presented on Fig. 7i and Supplementary Fig. 22a, and gating strategy to sort Tc (CD4-CD8+) and Th (CD4+CD8-) from T lymphocytes (CD3+CD49b+) for immune microenvironment analysis presented on Supplementary Fig. 22b. G1-G5 represent Control, Laser+GNR, Laser+hydrogel-GNR (1), Laser+hydrogel-GNR (2) and pNIPAAm, respectively.

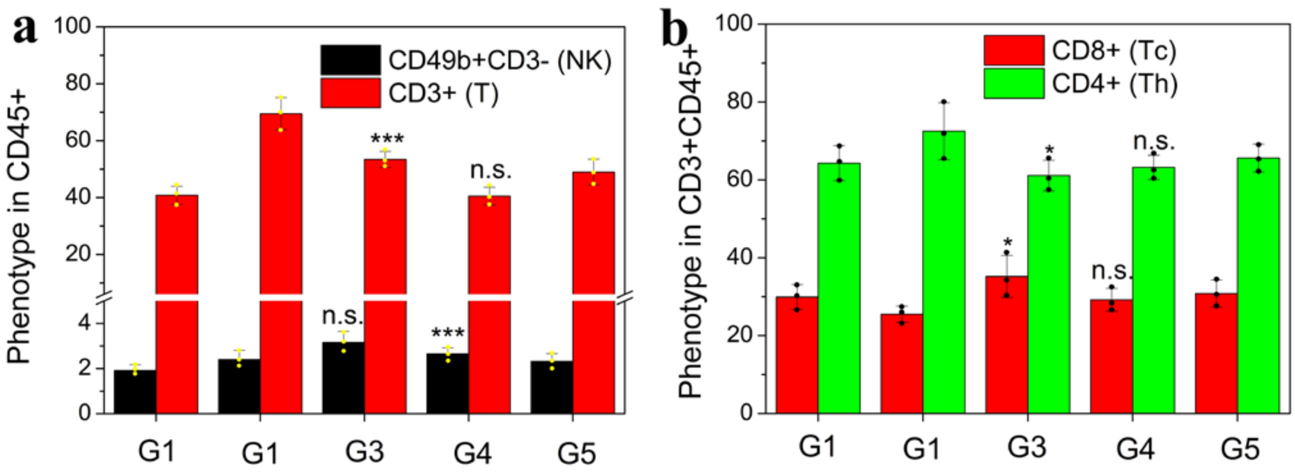

Supplementary Figure 22. (a) Relatively quantitative data of and NK cells (CD49b+CD3-) and T lymphocytes (CD3+) gating on in lymphocytes (CD45+) in tumor after different treatments (*i.e.*, G1-G5); (b) Relatively quantitative data of helper T (Th, CD4+) and cytotoxic T (Tc, CD8+) lymphocytes gating on in T lymphocytes (CD3+) in tumor after different treatments (*i.e.*, G1-G5). Data are expressed as mean ± SD (n = 3). Statistical significance was obtained using t-student test in comparison to G1. *P<0.05 and ***P < 0.001, and 'n.s.' means no statistical difference. G1-G5 represent Control, Laser+GNR, Laser+hydrogel-GNR (1), Laser+hydrogel-GNR (2) and pNIPAAm, respectively.

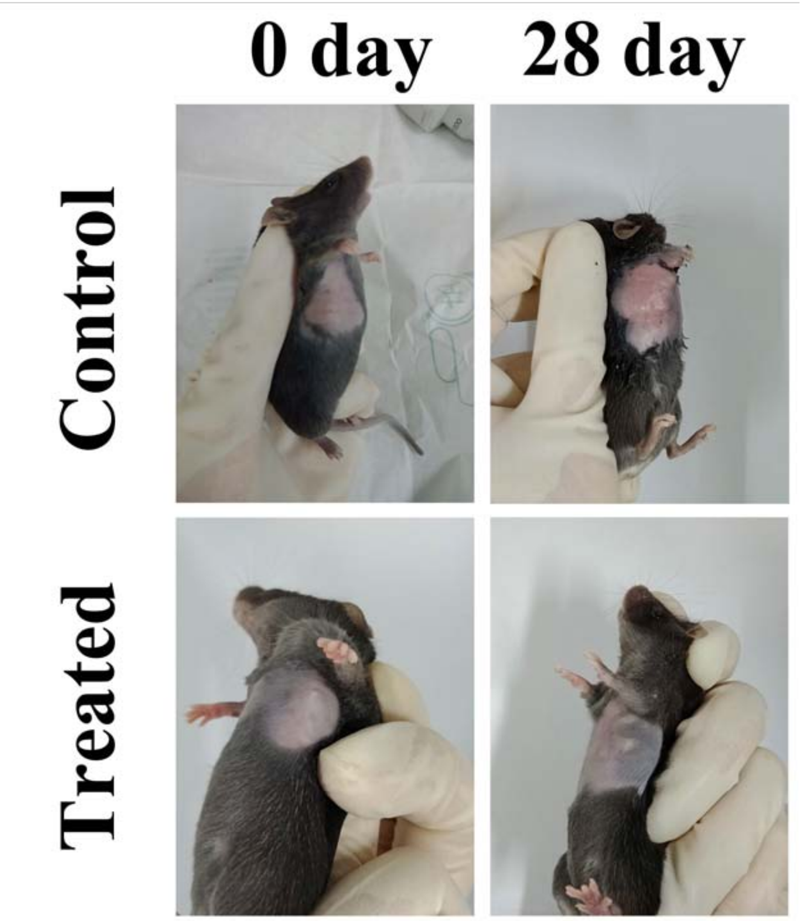

Supplementary Figure 23. Digital images of transgenic mice bearing MMTV-PyVmT oncogene-induced mammary adenocarcinomas in two groups (*i.e.*, control and treated) at 0 day and 28 day, respectively. In treated group, laser+hydrogel-GNR (2) treatment was carried out.

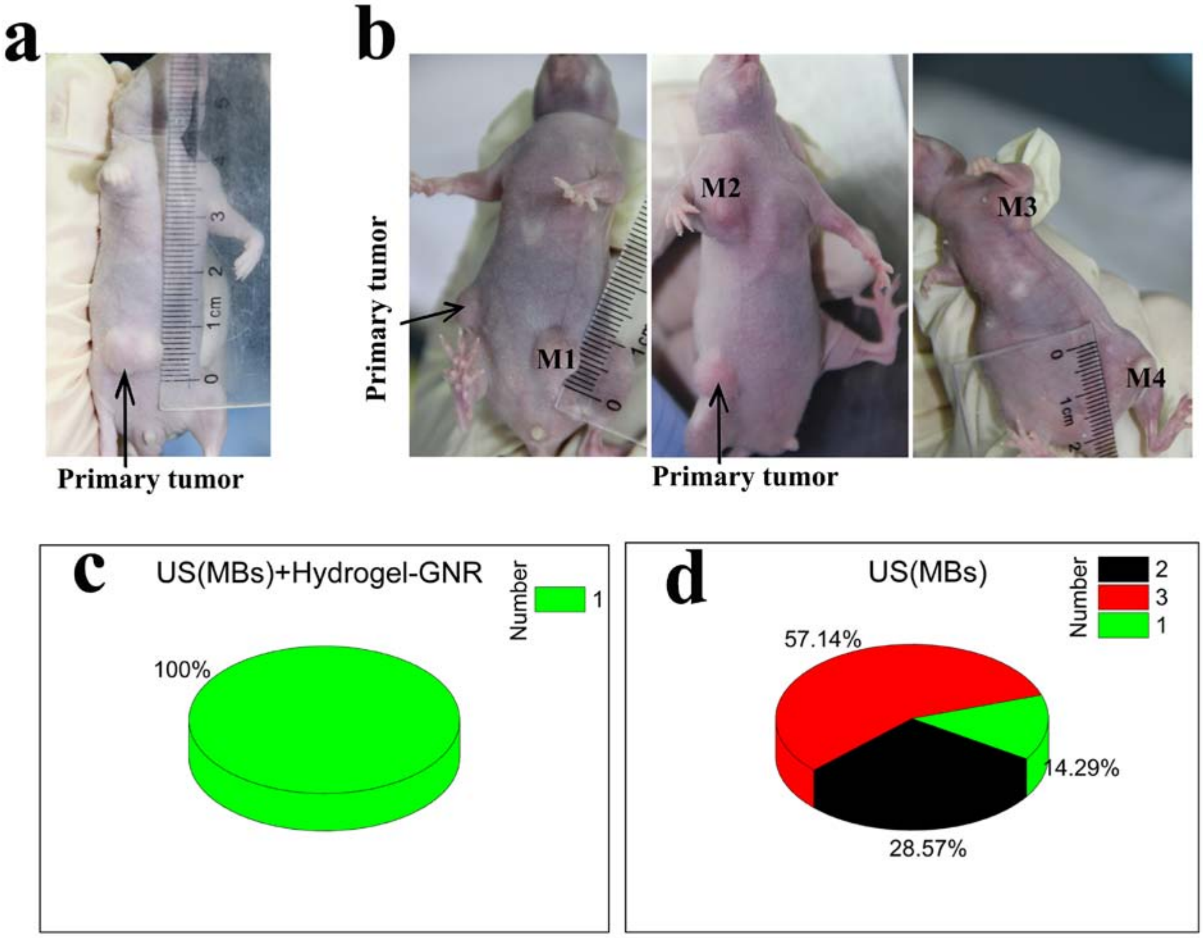

Supplementary Figure 24. (a,b) Digital photos of subcutaneously visible tumor variation in US(MBs)+hydrogel-GNR+Laser (a) and US(MBs) alone (b), wherein M1-M4 represent different distant metastasis sites and black arrows indicate tumor; (c,d) The statistically quantitative number of naked-eye tumors including primary tumor and distant metastasis in aforementioned two groups. Note: US(MBs)+hydrogel-GNR+Laser means that hydrogel-GNR injection and laser irradiation-mediated gelation were carried out on the primary tumor and its surrounding periphery, followed by US(MBs) treatment.

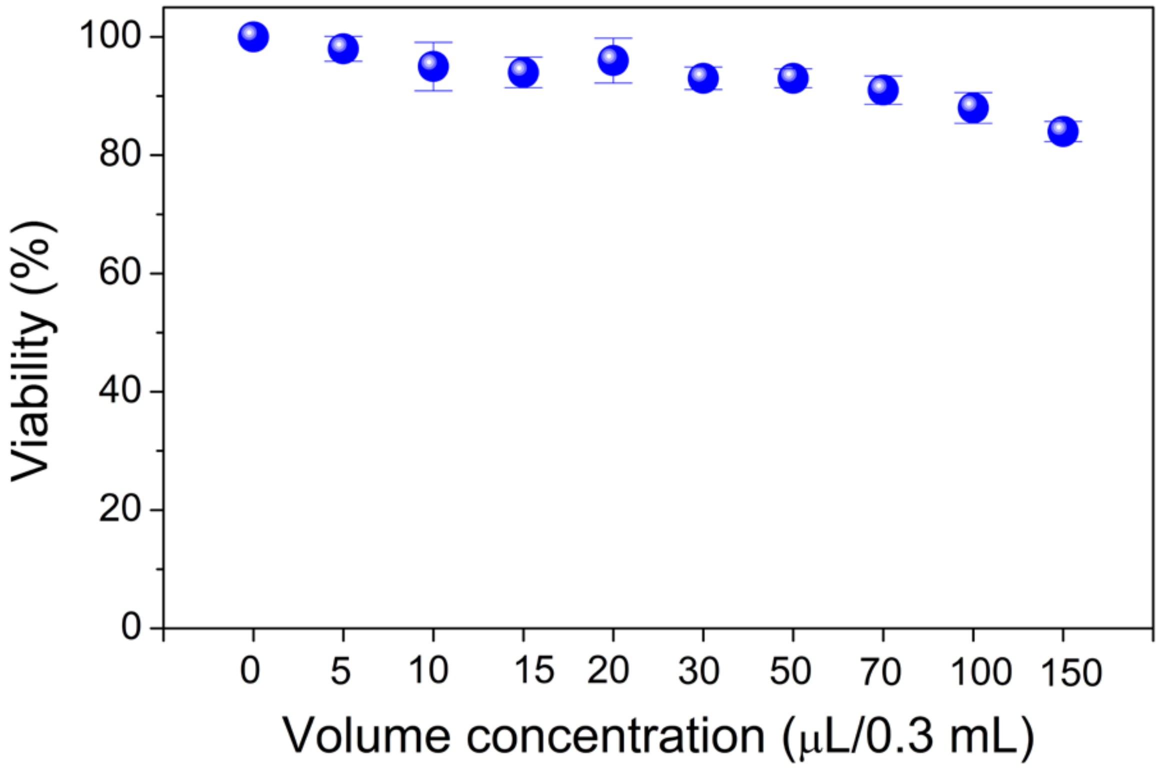

Supplementary Figure 25. Cell viability of PANC-1 cells after incubation with residual liquid of different concentrations obtained after gelation of hydrogel-GNR. Values are expressed as mean ± SD (n = 3).

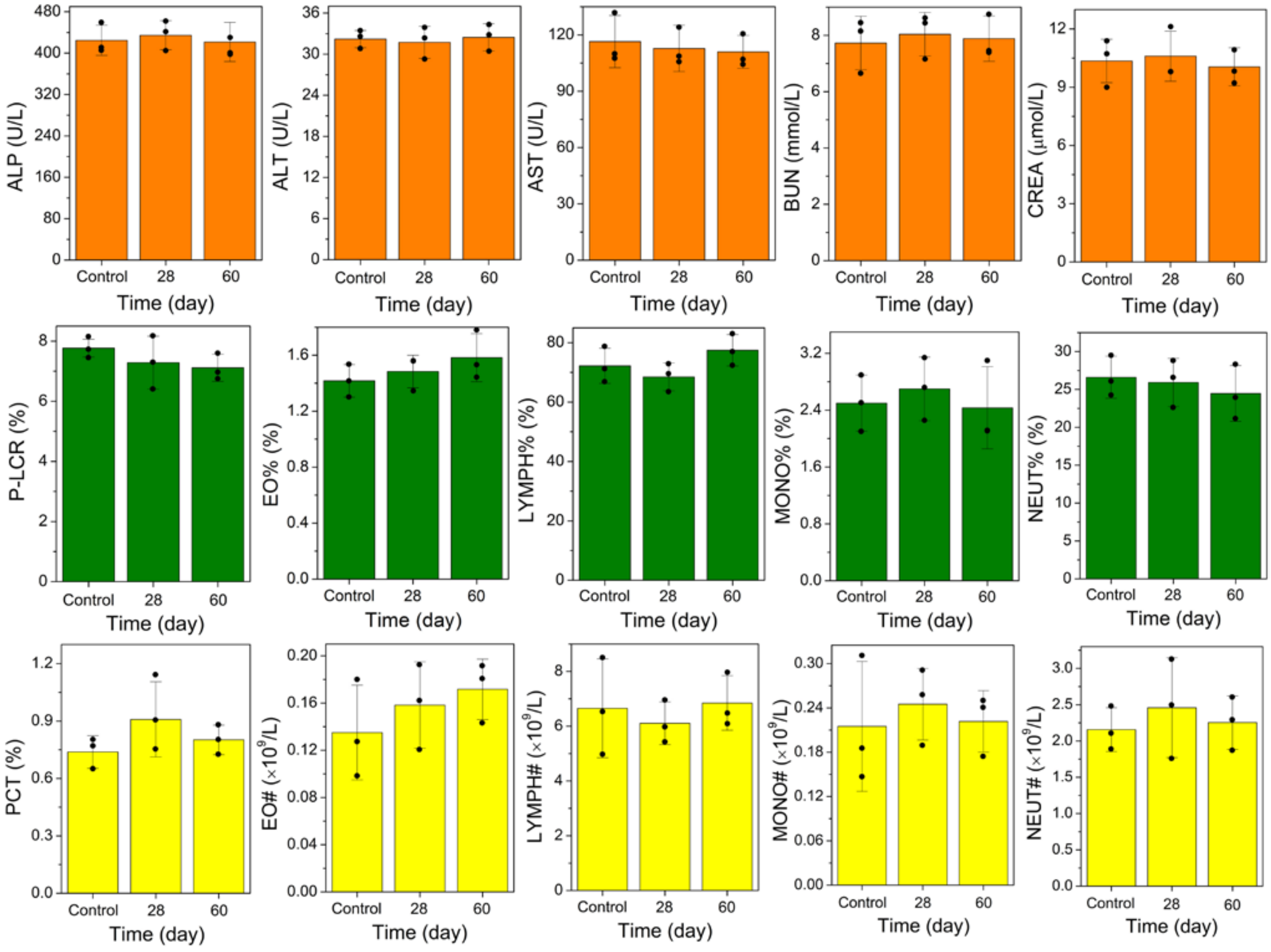

Supplementary Figure 26. Blood routine indexes of nude mice after intratumorally injecting hydrogel-GNR and laser irradiation (0.15 W·cm^{-2}) for 3 min in sum with a 30 s continuous laser irradiation and 10 s interval between two cycles, wherein the abdomen of Kunming mice received hydrogel-GNR injection and instant 808 nm laser irradiation. Values are expressed as mean ± SD (n = 3).

.

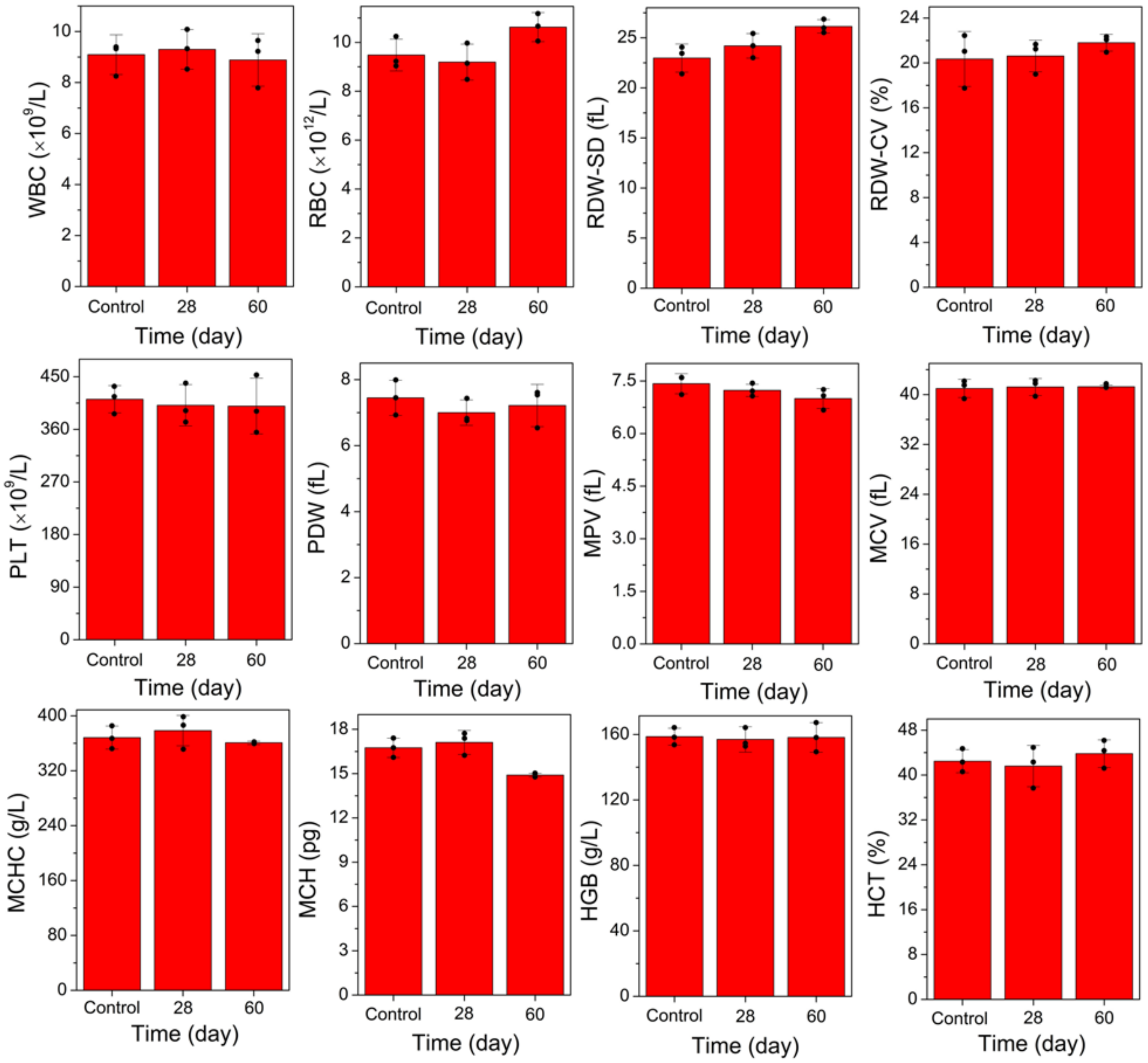

Supplementary Figure 27. Biochemical indexes of nude mice after intratumorally injecting hydrogel-GNR and laser irradiation (0.15 W·cm^{-2}) for 3 min in sum with a 30 s continuous laser irradiation and 10 s interval between two cycles, wherein the abdomen of Kunming mice received hydrogel-GNR injection and instant 808 nm laser irradiation. Values are expressed as mean ± SD (n = 3).

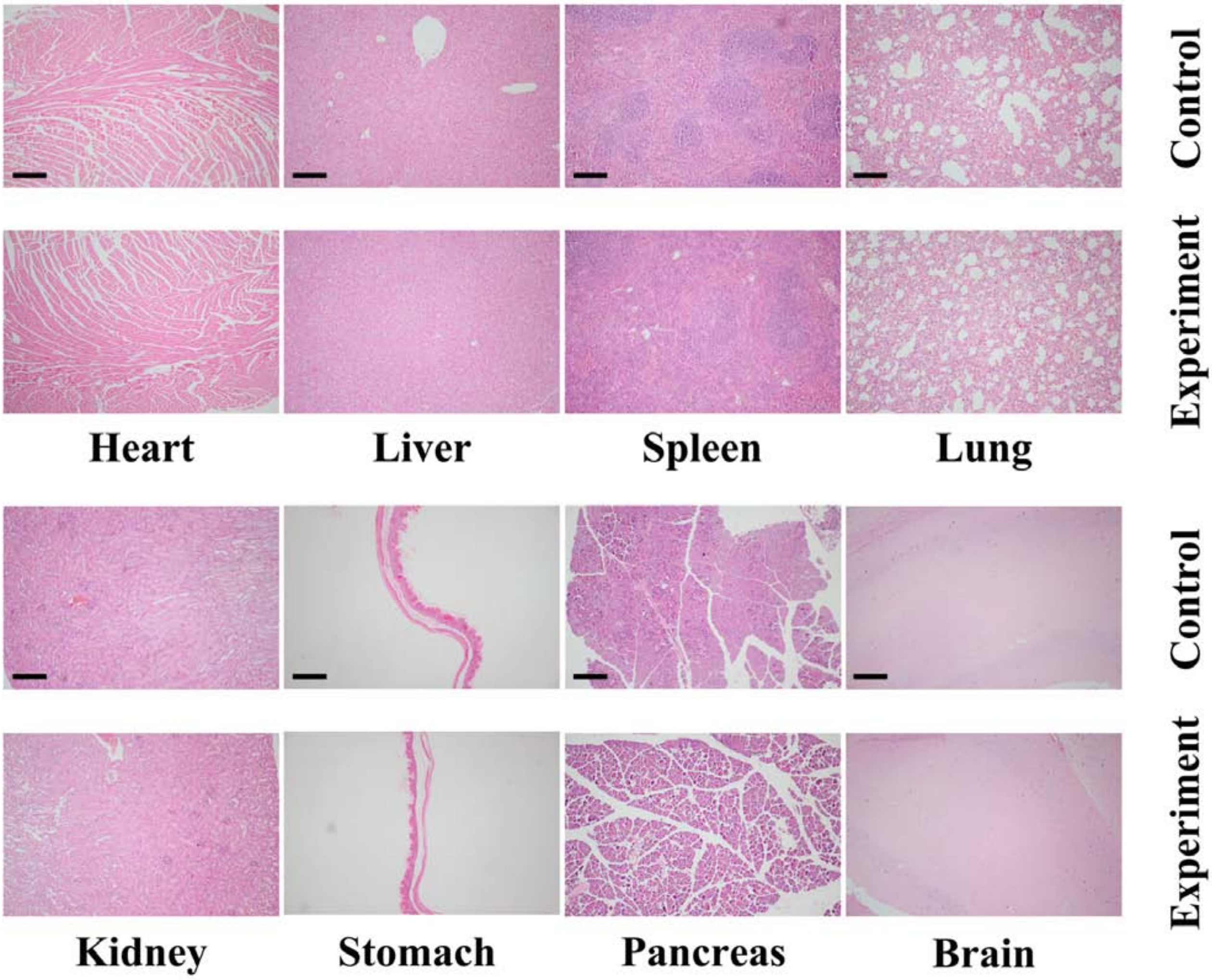

Supplementary Figure 28. Optical microscopic images of normal organs whose slices were stained by H&E immunohistochemical method (scale bar: 200 μm).

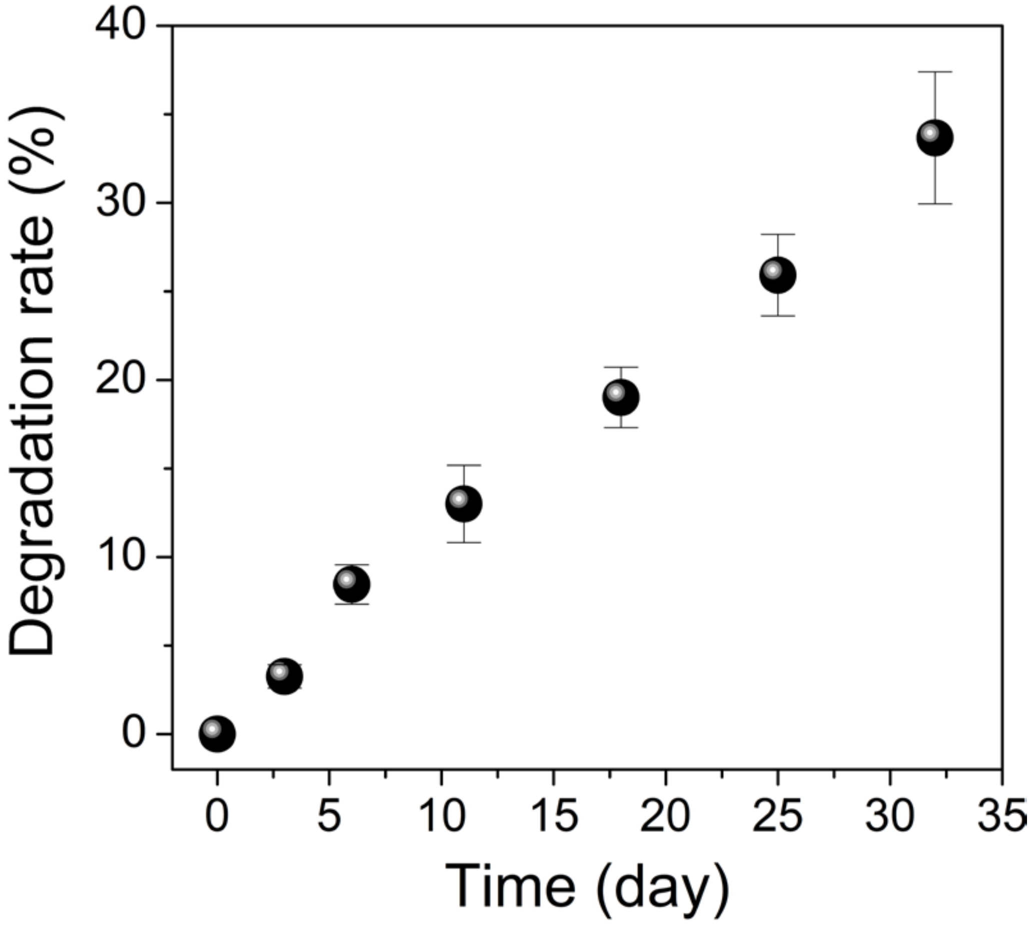

Supplementary Figure 29. *In vitro* degradation rate of hydrogel-GNR in buffer. Values are expressed as mean ± SD (n = 3).

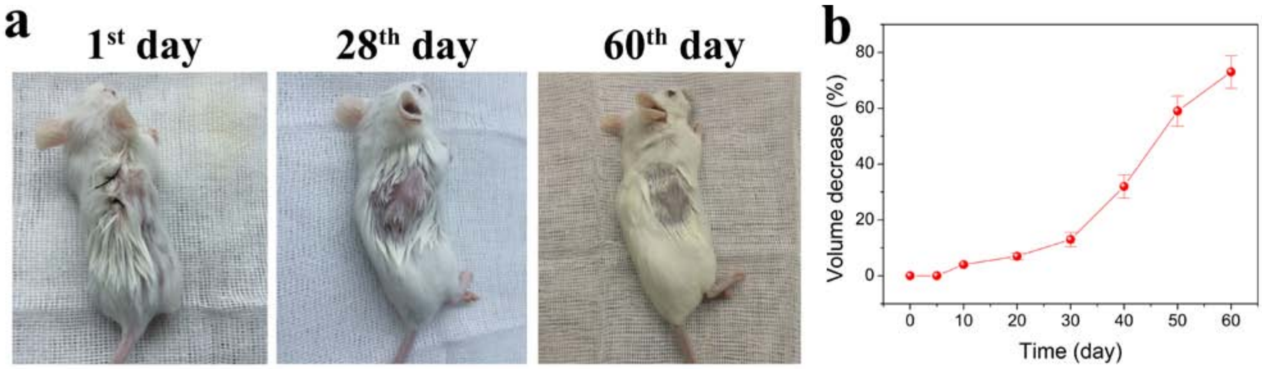

Supplementary Figure 30. (a) Digitals of *in vivo* degradation evaluation of hydrogel-GNR embedded in the abdomen of Kunming mice at different time points (1st day, 28th day and 60th day); (b) the quantitative volume decrease of hydrogel-GNR embedded in the abdomen of Kunming mice as a function of feeding time. Values are expressed as mean ± SD (n = 3).

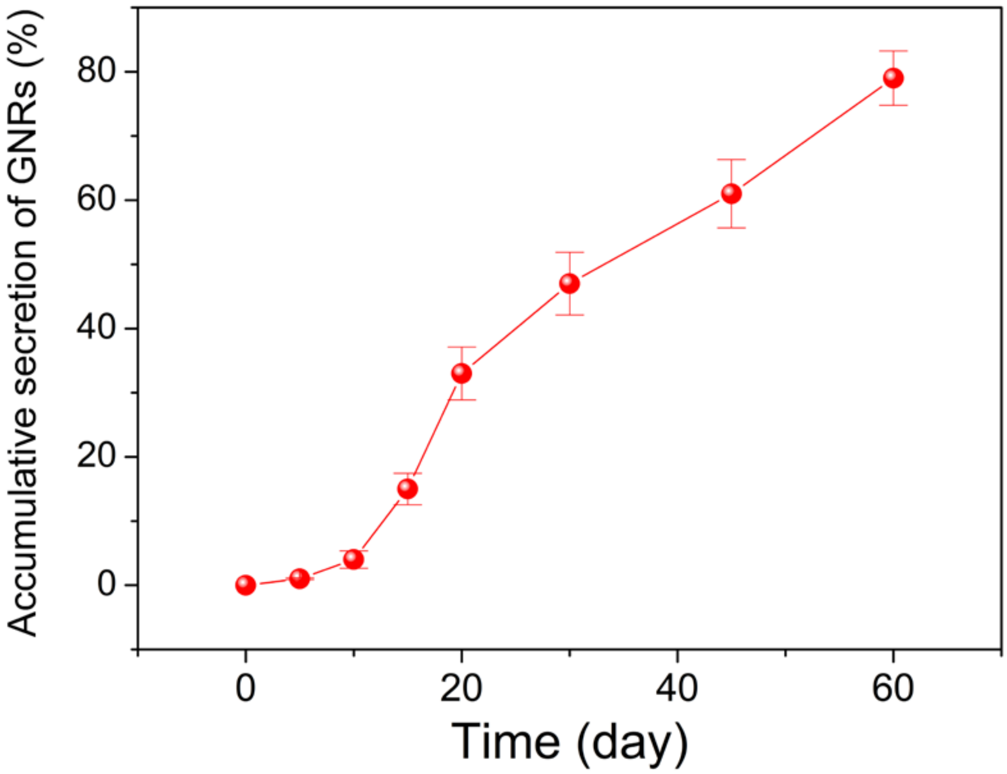

Supplementary Figure 31. Accumulative excretion level of GNRs in hydrogel-GNR after treatment with Laser+hydrogel-GNR (2) the as a function of time on PANC-1 tumor implanted on nude mice. Error bars are based on SD (n = 3);
