# Supplementary material for: Extravascular gelation shrinkage-derived internal stress enables tumor starvation therapy with suppressed metastasis and recurrence
Source: Nat Commun. 2019 Nov 26;10:5380. doi: 10.1038/s41467-019-13115-3 (PMC6879564; doi:10.1038/s41467-019-13115-3)
Supplement: Supplementary file 3 — Description of Additional Supplementary Files [file 41467_2019_13115_MOESM3_ESM.docx]

**Description of Additional Supplementary Files**

**File Name: Supplementary Movie 1**

**Description:** Color Doppler flow imaging (CDFI) movie of abdomen artery before treatment with hydrogel-GNR solution and 808 nm laser irradiation (Laser+hydrogel-GNR).

**File Name: Supplementary Movie 2**

**Description:** CDFI movie of abdomen artery after treatment with hydrogel-GNR solution and 808 nm laser irradiation (Laser+hydrogel-GNR).

**File Name: Supplementary Movie 3**

**Description:** CDFI movie of PANC-1 tumors implanted on nude mice before treatment with Laser+hydrogel-GNR (2).

**File Name: Supplementary Movie 4**

**Description:** CDFI movie of PANC-1 tumors implanted on nude mice after treatment with Laser+hydrogel-GNR (2).

**File Name: Supplementary Movie 5**

**Description:** CDFI movie of PANC-1 tumors implanted on nude mice after 5 days post-treatment with Laser+hydrogel-GNR (2).

**File Name: Supplementary Movie 6**

**Description:** CHI movie of PANC-1 tumor implanted on nude mice receiving the 1st injection of Sonovue microbubbles in control group.

**File Name: Supplementary Movie 7**

**Description:** CHI movie of PANC-1 tumor implanted on nude mice receiving the 2st injection of Sonovue microbubbles after 30-60 min post-treatment with corresponding treatment in control group.

**File Name: Supplementary Movie 8**

**Description:** CHI movie of PANC-1 tumor implanted on nude mice receiving the 1st injection of Sonovue microbubbles in treated group.

**File Name: Supplementary Movie 9**

**Description:** CHI movie of PANC-1 tumor implanted on nude mice receiving the 2st injection of Sonovue microbubbles after 30-60 min post-treatment with Laser+hydrogel-GNR (2) in treated group.

**File Name: Supplementary Movie 10**

**Description:** CHI movie of 4T1 tumor implanted on BALB/c mice receiving the 1st injection of Sonovue microbubbles in control group.

**File Name: Supplementary Movie 11**

**Description:** CHI movie of 4T1 tumor implanted on BALB/c mice receiving the 2st injection of Sonovue microbubbles after 30-60 min post-treatment with corresponding treatment in control group.

**File Name: Supplementary Movie 12**

**Description:** CHI movie of 4T1 tumor implanted on BALB/c mice receiving the 1st injection of Sonovue microbubbles in treated group.

**File Name: Supplementary Movie 13**

**Description:** CHI movie of 4T1 tumor implanted on BALB/c mice receiving the 2st injection of Sonovue microbubbles after 30-60 min post-treatment with Laser+hydrogel-GNR (2) in treated group.
