# Supplementary material for: Extravascular gelation shrinkage-derived internal stress enables tumor starvation therapy with suppressed metastasis and recurrence
Source: Nat Commun. 2019 Nov 26;10:5380. doi: 10.1038/s41467-019-13115-3 (PMC6879564; doi:10.1038/s41467-019-13115-3)
Supplement: Supplementary file 17 — Reporting Summary [file 41467_2019_13115_MOESM17_ESM.pdf]

natureresearch

Corresponding author(s): Kun Zhang

Last updated by author(s): Apr 23, 2019

Reporting Summary

Nature Research wishes to improve the reproducibility of the work that we publish. This form provides structure for consistency and transparency in reporting. For further information on Nature Research policies, see Authors & Referees and the Editorial Policy Checklist .

Statistics

For all statistical analyses, confirm that the following items are present in the figure legend, table legend, main text, or Methods section.

n/a	Confirmed	
☐	☒	The exact sample size (*n*) for each experimental group/condition, given as a discrete number and unit of measurement
☐	☒	A statement on whether measurements were taken from distinct samples or whether the same sample was measured repeatedly
☐	☒	The statistical test(s) used AND whether they are one- or two-sided<br>*Only common tests should be described solely by name; describe more complex techniques in the Methods section.*
☐	☒	A description of all covariates tested
☐	☒	A description of any assumptions or corrections, such as tests of normality and adjustment for multiple comparisons
☐	☒	A full description of the statistical parameters including central tendency (e.g. means) or other basic estimates (e.g. regression coefficient) AND variation (e.g. standard deviation) or associated estimates of uncertainty (e.g. confidence intervals)
☐	☒	For null hypothesis testing, the test statistic (e.g. *F*, *t*, *r*) with confidence intervals, effect sizes, degrees of freedom and *P* value noted<br>*Give P values as exact values whenever suitable.*
☒	☐	For Bayesian analysis, information on the choice of priors and Markov chain Monte Carlo settings
☒	☐	For hierarchical and complex designs, identification of the appropriate level for tests and full reporting of outcomes
☒	☐	Estimates of effect sizes (e.g. Cohen's *d*, Pearson's *r*), indicating how they were calculated

Our web collection on statistics for biologists contains articles on many of the points above.

Software and code

Policy information about availability of computer code

Data collection	Excel, CytExpert software, FV10-ASW 1.7 Viewer, ImageJ 1.43u
Data analysis	All statistical analyses were performed on Origin software (8.1), Excel, and Graphpad Prism (version 7). All flow cytometry data were analyzed on FlowJo software package (version 10.0.7; TreeStar, USA, 2014). Living image software (Perkin Elmer) was used to analyse bioluminescent and fluorescent images. The average sound intensity values can be obtained from an image processing software, SONOMATHdDICOM. MRI image data were captured on ImageJ 1.43u.

For manuscripts utilizing custom algorithms or software that are central to the research but not yet described in published literature, software must be made available to editors/reviewers. We strongly encourage code deposition in a community repository (e.g. GitHub). See the Nature Research guidelines for submitting code & software for further information.

Data

Policy information about availability of data

All manuscripts must include a data availability statement. This statement should provide the following information, where applicable:

- Accession codes, unique identifiers, or web links for publicly available datasets
- A list of figures that have associated raw data
- A description of any restrictions on data availability

A list of figures that have associated raw data.

Field-specific reporting

Please select the one below that is the best fit for your research. If you are not sure, read the appropriate sections before making your selection.

Life sciences study design

All studies must disclose on these points even when the disclosure is negative.

Sample size	No effect size was predetermined. All the biochemical and biological experiments were performed in three replicated or more.
Data exclusions	No data were excluded.
Replication	Experiments were repeated and experimental findings were reproducible
Randomization	Mice were allocated randomly to each group.
Blinding	All experimental procedures and quantification of results were done by two independent researchers.

Reporting for specific materials, systems and methods

We require information from authors about some types of materials, experimental systems and methods used in many studies. Here, indicate whether each material, system or method listed is relevant to your study. If you are not sure if a list item applies to your research, read the appropriate section before selecting a response.

Materials & experimental systems

n/a	Involved in the study	
☐	☒	Antibodies
☐	☒	Eukaryotic cell lines
☒	☐	Palaeontology
☐	☒	Animals and other organisms
☒	☐	Human research participants
☒	☐	Clinical data

Methods

n/a	Involved in the study	
☒	☐	ChIP-seq
☐	☒	Flow cytometry
☐	☒	MRI-based neuroimaging

Antibodies

Antibodies used

The following primary antibodies were used for western blotting. They are listed as antigen first, followed by supplier and catalog number as applicable.
1) Anti-bcl-2 antibody, Abcam, cat. no. ab32124,
2) Anti caspase-3 antibody, Abcam, cat. no. ab13847;
3) Anti-HSP70 antibody, Abcam, cat. no. ab2787;
4) Anti-p53 antibody, Abcam, cat. no. ab131442;
5) Anti-CD34 antibody, Abcam, cat. no. ab8158;
6) Anti-CD31 antibody, Abcam, cat. no. ab28364;
7) Anti-beta Actin Antibody, Abcam, cat. no. ab6276;
8) Anti-HIF-1 alpha antibody, Abcam, cat. no. ab16066
The following primary antibodies were used for immunofluorescence. They are listed as antigen first, followed by supplier and catalog number as applicable.
1) Anti-mouse CD34, Abcam, cat. no. ab8158;
2) Anti-mouse CD31, Abcam, cat. no. ab24590;
3) DAPI, Beyotime Biotechnology, cat. no. C1002;
4) TUNEL apoptotic assay kit, Roche Applied Science, cat. no. 11684817910
The following primary antibodies were used for immunohistochemistry. They are listed as antigen first, followed by supplier and catalog number as applicable.
1) Anti-mouse PCNA, Abcam, cat. no. ab29
2) Anti-CD34 antibody, Abcam, cat. no. ab8158
3) Hematoxylin and Eosin Staining Kit, Beyotime Biotechnology, cat. no. C0105
The following primary antibodies were used for flow cytometry. They are listed as antigen first, followed by supplier, catalog number and clone/lot number as applicable.
1) Anti-mouse CD45, Biolegend, cat. no. 103108, Clone: 30-F11;
2) Anti-mouse CD11b, Biolegend, cat. no. 101208, Clone: M1/70;
3) Anti-mouse F4/80, Biolegend, cat. no. 123116, Clone:BM8;
4) Anti-mouse CD49b Antibody, cat. no. 103516 , Clone: HMα2
5) Anti-mouse CD3, Biolegend, cat. no. 100204， Clone: 17A2;
6) Anti-mouse CD4, Biolegend, cat. no. 100412, Clone: GK1.5;
7) Anti-mouse CD8, Biolegend, cat. no. 140408, Clone: 53-5.8;
8) Anti-mouse CD11c, Biolegend, cat. no. 117310, Clone: N418;

The following primary antibodies were used for ELISA. They are listed as antigen first, followed by supplier, catalog number and clone/lot number as applicable.
1) Ani-mouse IL-1β, Invitrogen, at. no. MM425B
1) Anti-mouse IL-4, eBioscience, cat. no. 14-7041-81;
2) Anti-mouse IL-5, Invitrogen, cat. no. MM550CB;
3) Anti-mouse IL-13, eBioscience, cat. no. 14-7133-81;
4) Anti-mouse IL-17, eBioscience, cat. no. 13-7177-81;
5) Anti-mouse IL-18, Invitrogen, cat. no. PA5-81413;
6) Anti-rat IL-21, Invitrogen, cat. no. MA5-30812;
7) Anti-mouse IL-23, eBioscience, cat. no. 13-7123-81;
8) Anti-mouse IL-6, eBioscience, cat. no. 14-7061-81;
9) Anti-mouse IFN gamma, Invitrogen, cat. no. BMS606INST;
10) Anti-mouse TNF α, eBioscience, cat. no. 14-7325-81;
11) Anti-mouse TNF β, eBioscience, cat. no. BMS105;
12) Anti-mouse IL-8, Invitrogen, cat. no. M801;
13) Anti-mouse IL-12, eBioscience, cat. no. 16-7101-81;
14) Anti-mouse CCL-22, Invitrogen, cat. no. MA5-23780;
15) Anti-mouse CCL-17, Invitrogen, cat. no. PA5-78933;
16) Anti-mouse CCL-5, eBioscience, cat. no. 14-7085-82

Validation: All antibodies were verified by the supplier and each lot has been quality tested.

Eukaryotic cell lines

Policy information about cell lines

Cell line source(s): The human pancreatic carcinoma cell line PANC-1 and mice breast carcinoma cell line 4T1 were purchased from Shanghai Institutes for Biological Sciences, Shanghai, China.

Authentication: Cell lines authentication was performed by short tandem repeat DNA profiling and comparison with reference database.

Mycoplasma contamination: All cell lines were tested for mycoplasma contamination. No mycoplasma contamination was found.

Commonly misidentified lines (See ICLAC register): No commonly misidentified cell lines were used.

Animals and other organisms

Policy information about studies involving animals; ARRIVE guidelines recommended for reporting animal research

Laboratory animals: Balb/c nu/nu nude mice (6–10 weeks), Kunming mice female BALB/c mice and were purchased from Shanghai Xipuer-Bikai Laboratory Animal Technology Co., Ltd.. Female transgenic mice were purchased from both Shanghai Hanyin biotech. Co.Ltd, and Nanning Wilking Biotechnology Co., Ltd.

Wild animals: The study did not involve wild animals.

Field-collected samples: The study did not involve samples collected from field.

Ethics oversight: All experiments the experiments were performed according to protocols approved by the Laboratory Animal Center of Shanghai Tenth Peoples' Hospital and were in accordance with the policies of National Ministry of Health.

Note that full information on the approval of the study protocol must also be provided in the manuscript.

Flow Cytometry

Plots

Confirm that:

- [x] The axis labels state the marker and fluorochrome used (e.g. CD4-FITC).
- [x] The axis scales are clearly visible. Include numbers along axes only for bottom left plot of group (a 'group' is an analysis of identical markers).
- [x] All plots are contour plots with outliers or pseudocolor plots.
- [x] A numerical value for number of cells or percentage (with statistics) is provided.

Methodology

Sample preparation	For tissue sample, the tissue was first mechanically disrupted from mice and divided into small pieces and homogenized in cold staining buffer to form single cell suspensions in the presence of digestive enzyme.
Instrument	CytoFLEX flow cytometer (Beckman)
Software	FlowJo software package (version 10.0.7; TreeStar, USA, 2014)
Cell population abundance	No sorting was performed.
Gating strategy	Generally, cells was first gated on SSC-A. Singlet cells were usually gated using FSC-H and FSC-A. Surface antigen gating was performed on the live cell population.

[x] Tick this box to confirm that a figure exemplifying the gating strategy is provided in the Supplementary Information.

Magnetic resonance imaging

Experimental design

Design type	Magnetic resonance imaging (MRI) was used to assess diffusion of Fe3O4 nanoparticles after i.t. administering into PANC-1 tumor implanted on nude mice.
Design specifications	Not applicable
Behavioral performance measures	Not applicable

Acquisition

Imaging type(s)	Structural MRI
Field strength	3.0 Tesla
Sequence & imaging parameters	T2-TSE-Tra sequence: TR = 1740 ms, TE1 = 4.36 ms, TE2 = 11.90 ms, TE3 = 19.44 ms, TE4 = 26.98 ms, TE5 = 34.52 ms, BW = 260 Hz, Thickness = 2 mm, Slices = 20, FOV = 200*200.
Area of acquisition	Axial planes thoracic cavity scan

Diffusion MRI [ ] Used [x] Not used

Preprocessing

Preprocessing software	Not applicable
Normalization	Not applicable
Normalization template	Not applicable
Noise and artifact removal	Not applicable
Volume censoring	Not applicable

Statistical modeling & inference

Model type and settings	Not applicable
Effect(s) tested	Not applicable

Specify type of analysis: [ ] Whole brain [x] ROI-based [ ] Both

Anatomical location(s): Tumor

Statistic type for inference (See Eklund et al. 2016)	Not applicable
Correction	Not applicable

Models & analysis

n/a	Involved in the study
☒	☐ Functional and/or effective connectivity
☒	☐ Graph analysis
☒	☐ Multivariate modeling or predictive analysis
